# Supplementary figures and images for: Unsupervised AI reveals insect species-specific genome signatures
Source: PeerJ. 2024 Mar 6;12:e17025. doi: 10.7717/peerj.17025 (PMC10924456; doi:10.7717/peerj.17025)

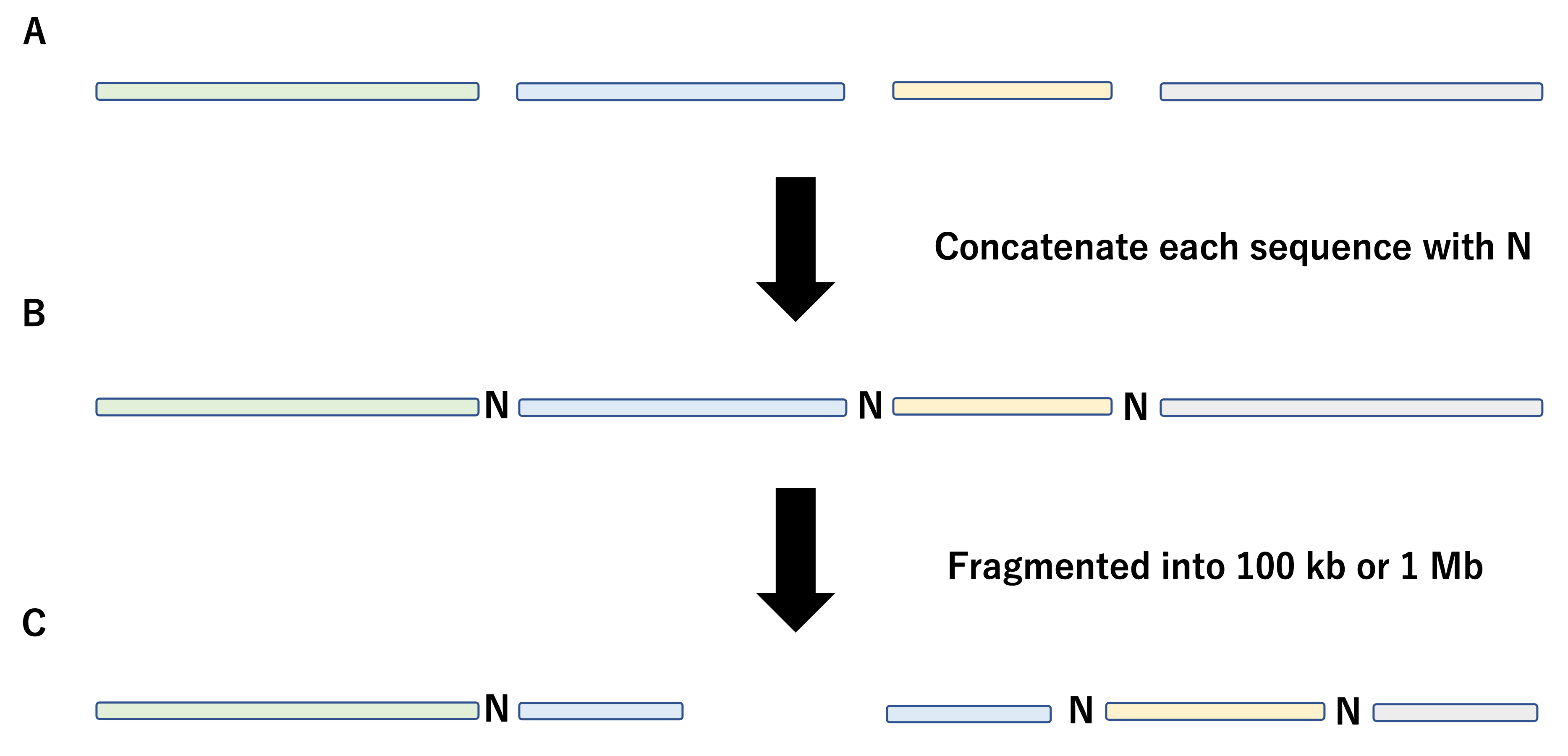

Supplement: Figure S1 — Short sequences (A) were joined by adding an N. After concatenation of all short sequences (B), the concatenated sequence was used for analysis after segmentation into 1-Mb or 100-kb sequences (C). [file peerj-12-17025-s001.png]

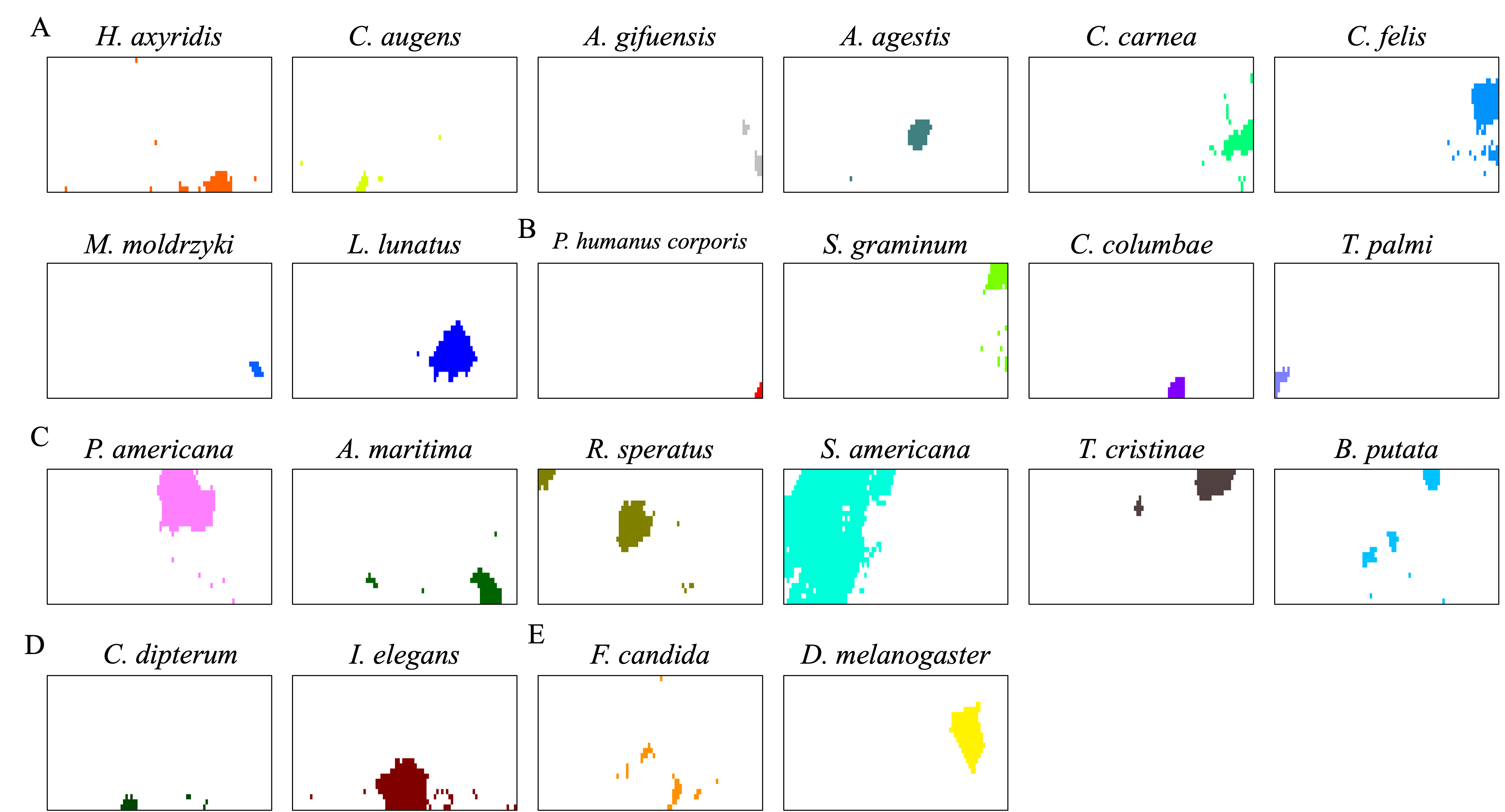

Supplement: Figure S2 — Nodes containing sequences from each species are colored as described in Figure 1A. [file peerj-12-17025-s002.png]

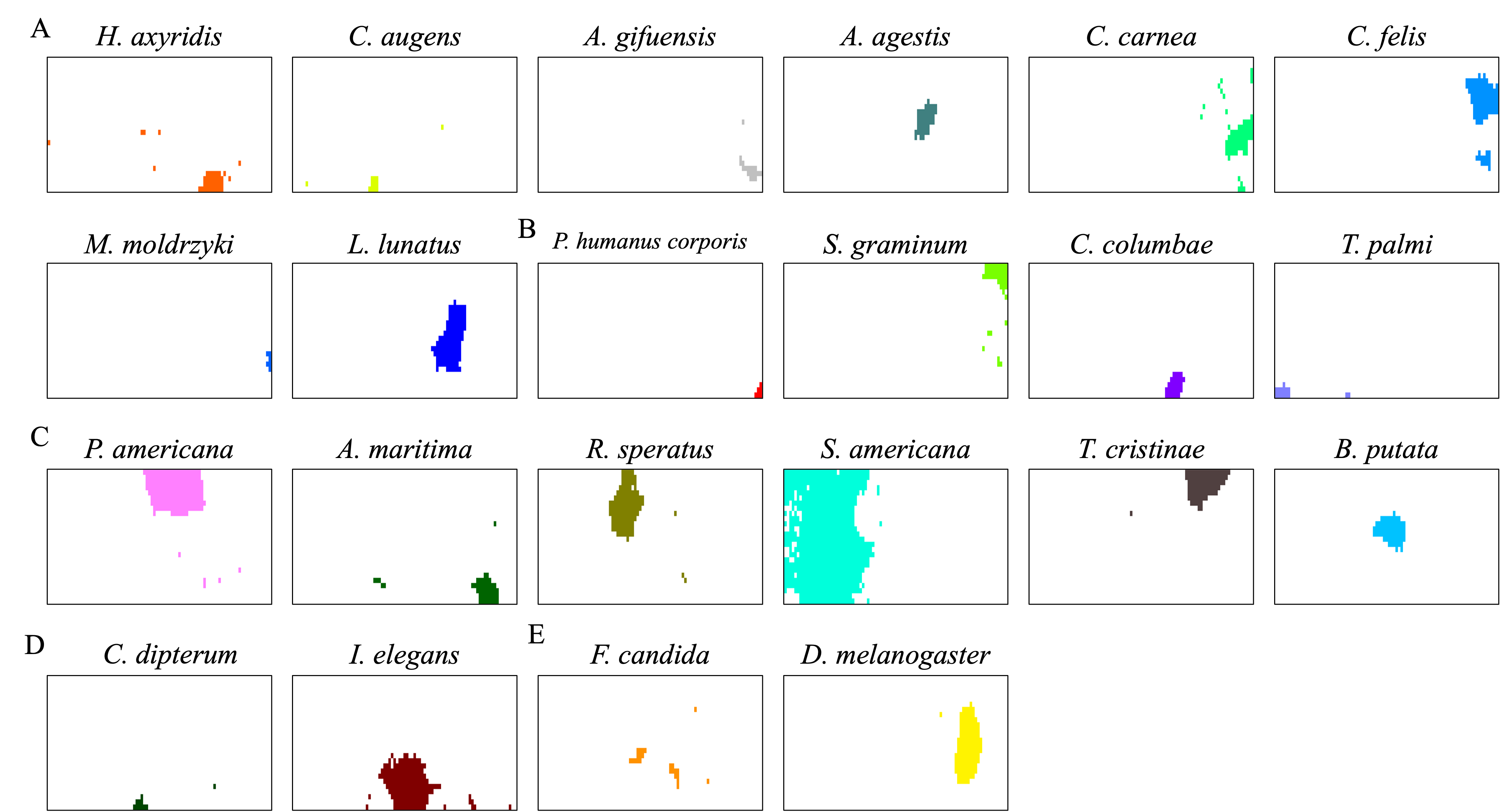

Supplement: Figure S3 — Nodes containing sequences from each species are colored as described in Figure 1A. [file peerj-12-17025-s003.png]

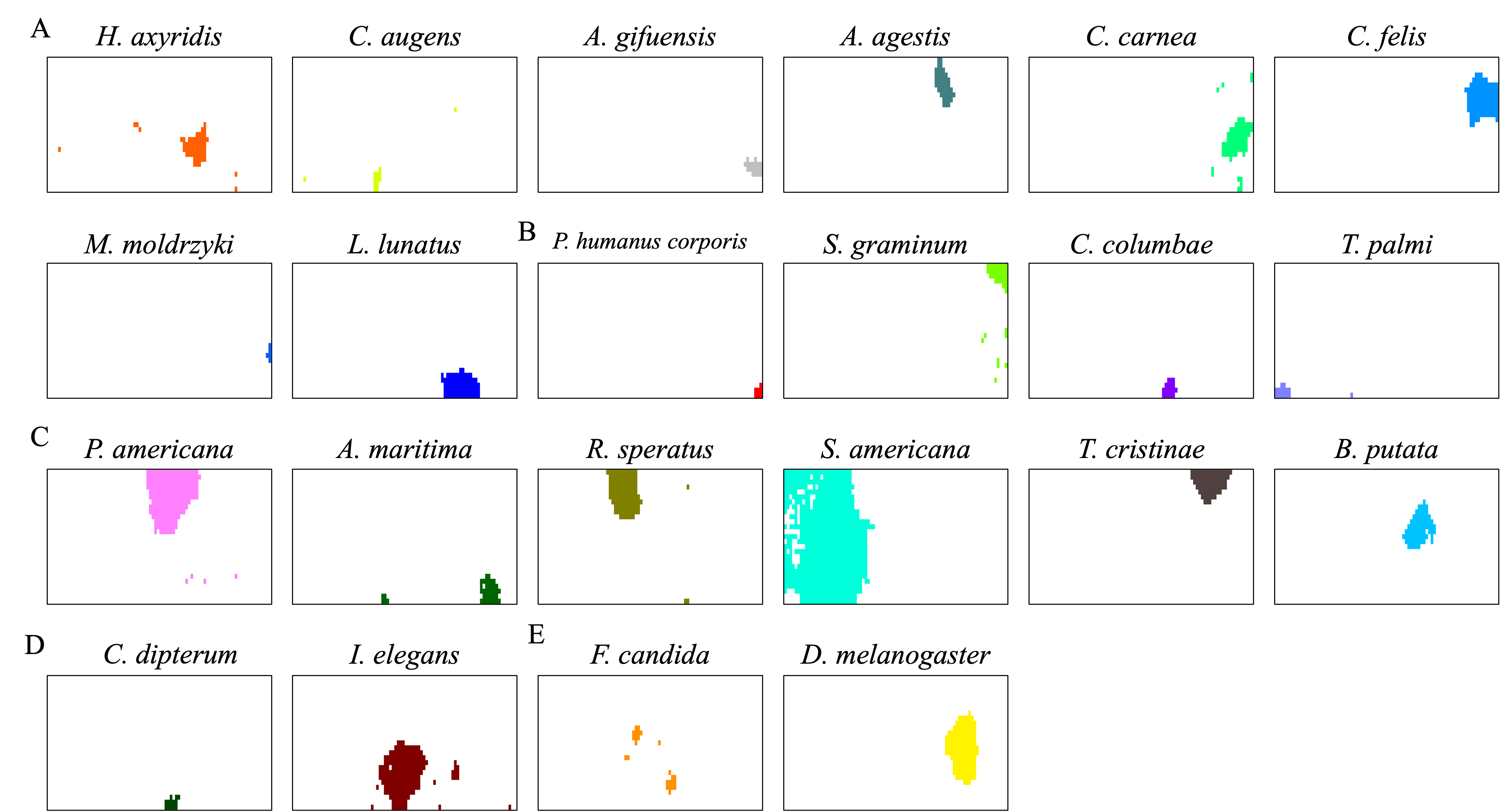

Supplement: Figure S4 — Nodes containing sequences from each species are colored as described in Figure 1A. [file peerj-12-17025-s004.png]

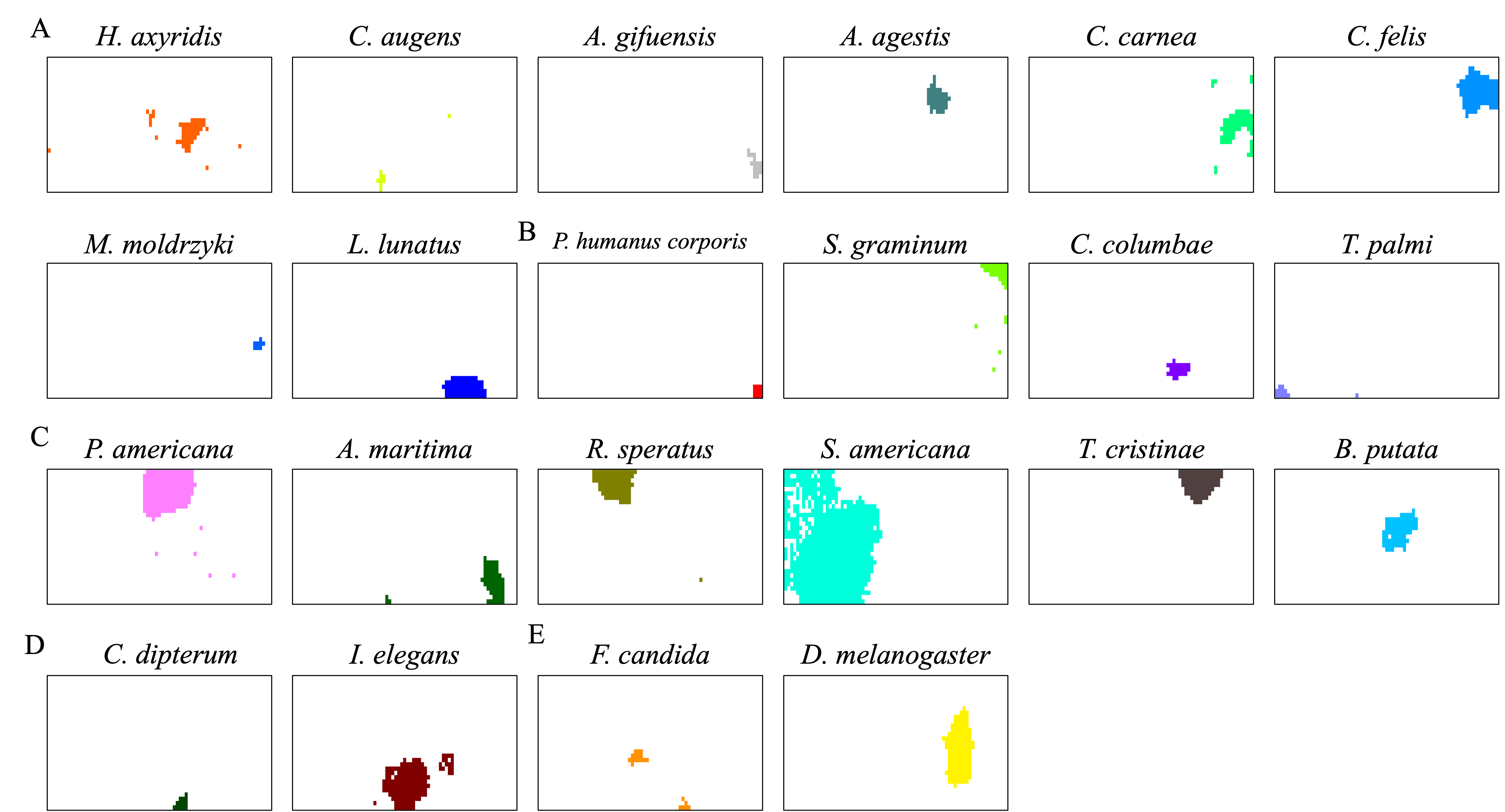

Supplement: Figure S5 — Nodes containing sequences from each species are colored as described in Figure 1A. [file peerj-12-17025-s005.png]

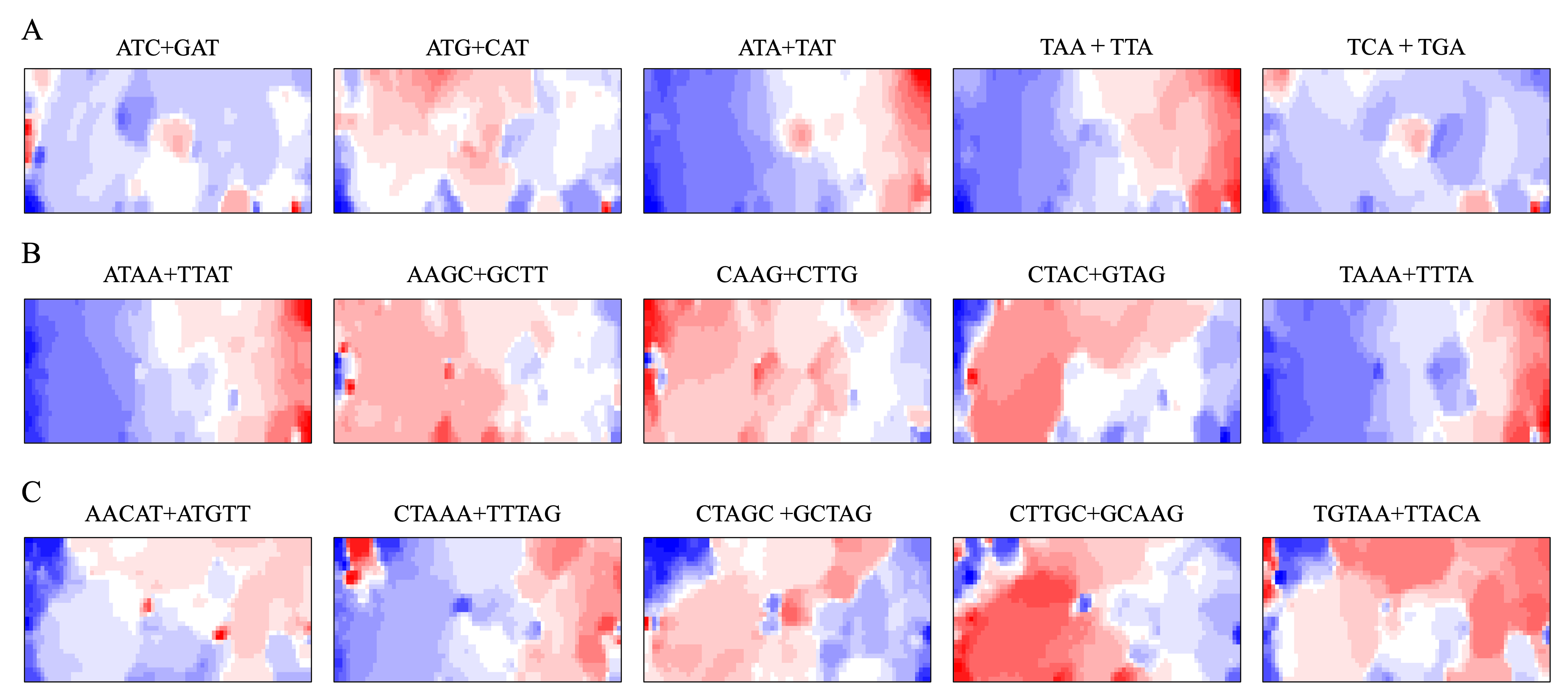

Supplement: Figure S6 — (A) DegeTri, (B) DegeTetra, and (C) DegePenta. The contribution of each oligonucleotide at each node is visualized by color: dark red (very high), red (high), white (moderate), blue (low), and dark blue (very low). Orange/blue heatmap patterns were also presented, for the easy accessibility to those with non-normal color vision as Figure S18. [file peerj-12-17025-s006.png]

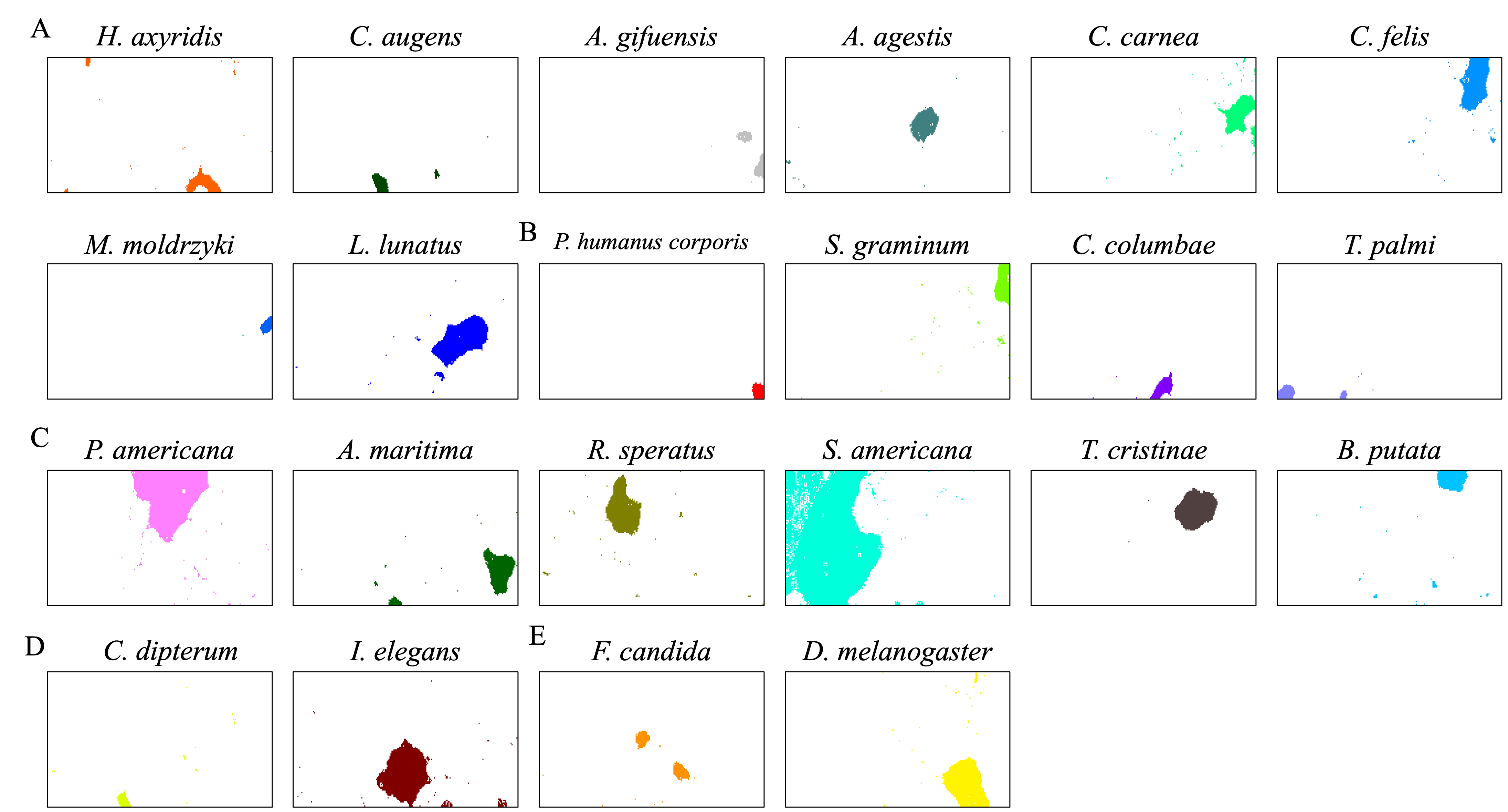

Supplement: Figure S7 — Nodes containing sequences from each species are colored as described in Figure 1A. [file peerj-12-17025-s007.png]

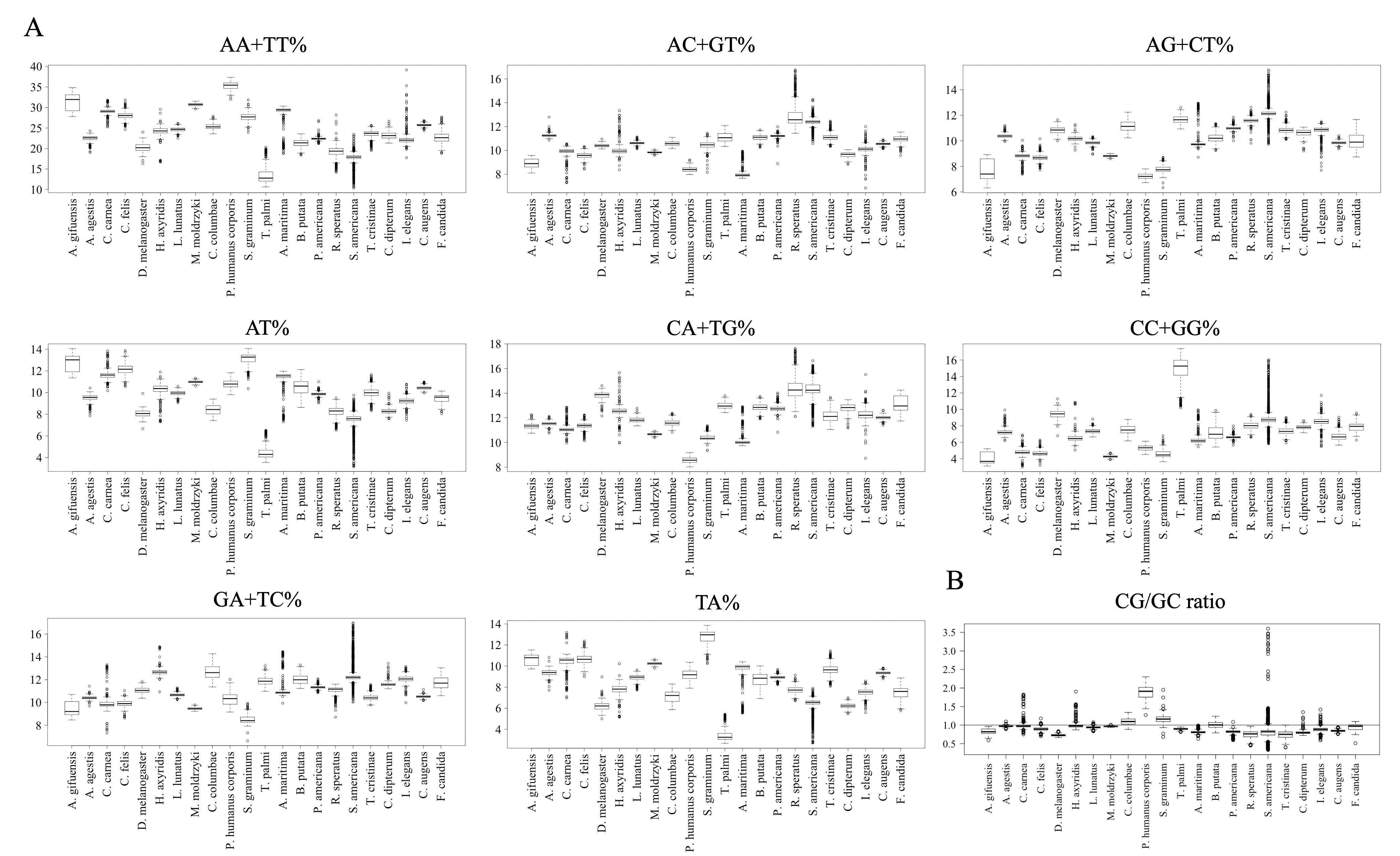

Supplement: Figure S8 — Boxplots showing occurrence (A) frequencies (%) of each dinucleotide, (B) CG/GC ratio in 1-Mb fragments of each insect. [file peerj-12-17025-s008.png]

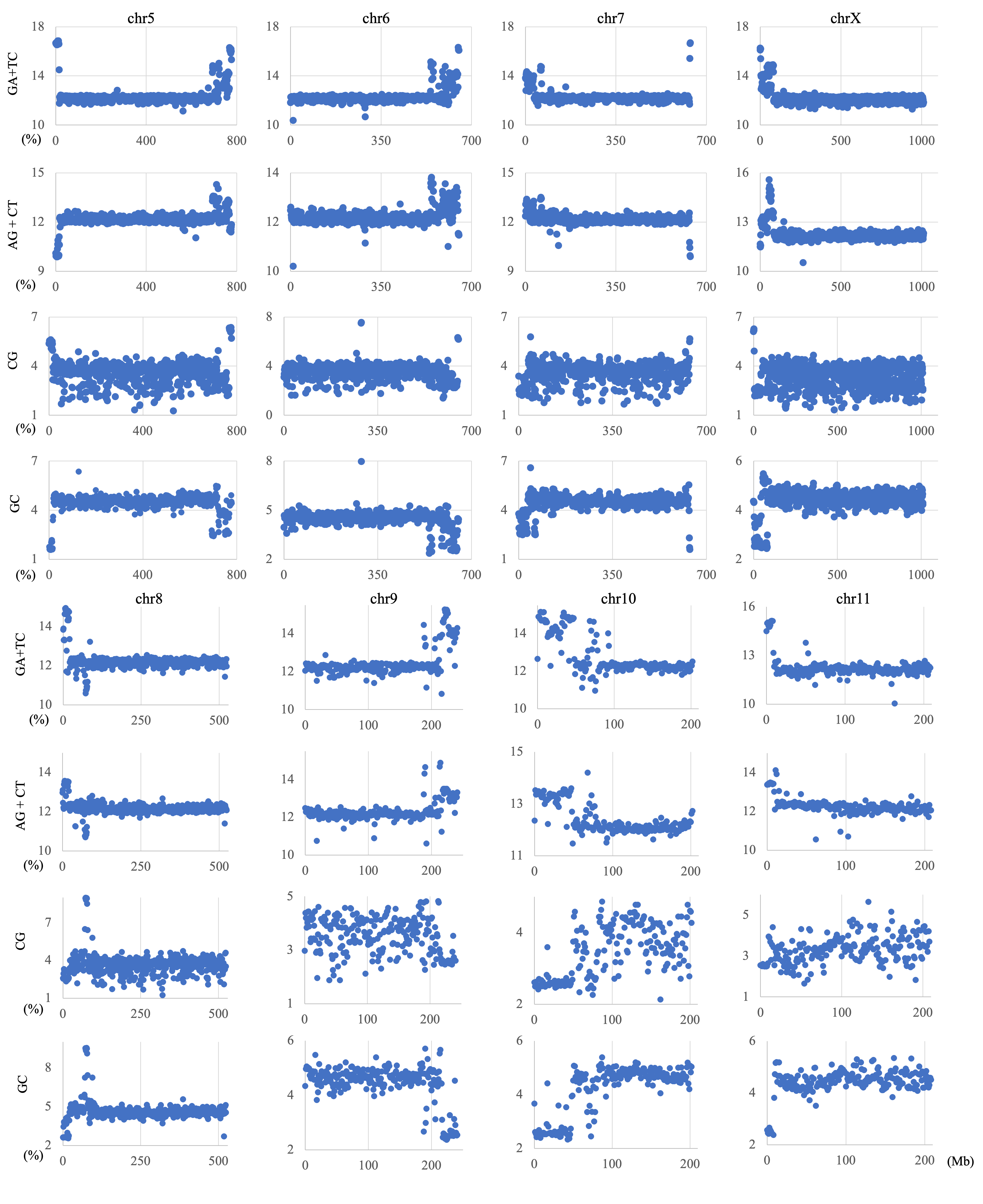

Supplement: Figure S9 — Distribution charts showing chromosomal distribution of GA + TC, AG + CT, CG, or GC on chromosomes 5, 6, 7, 8, 9, 10, 11, and X in 1 Mb sequences of S. amerinaca are presented. The vertical axis represents the occurrence frequency (%) of each dinucleotide. [file peerj-12-17025-s009.png]

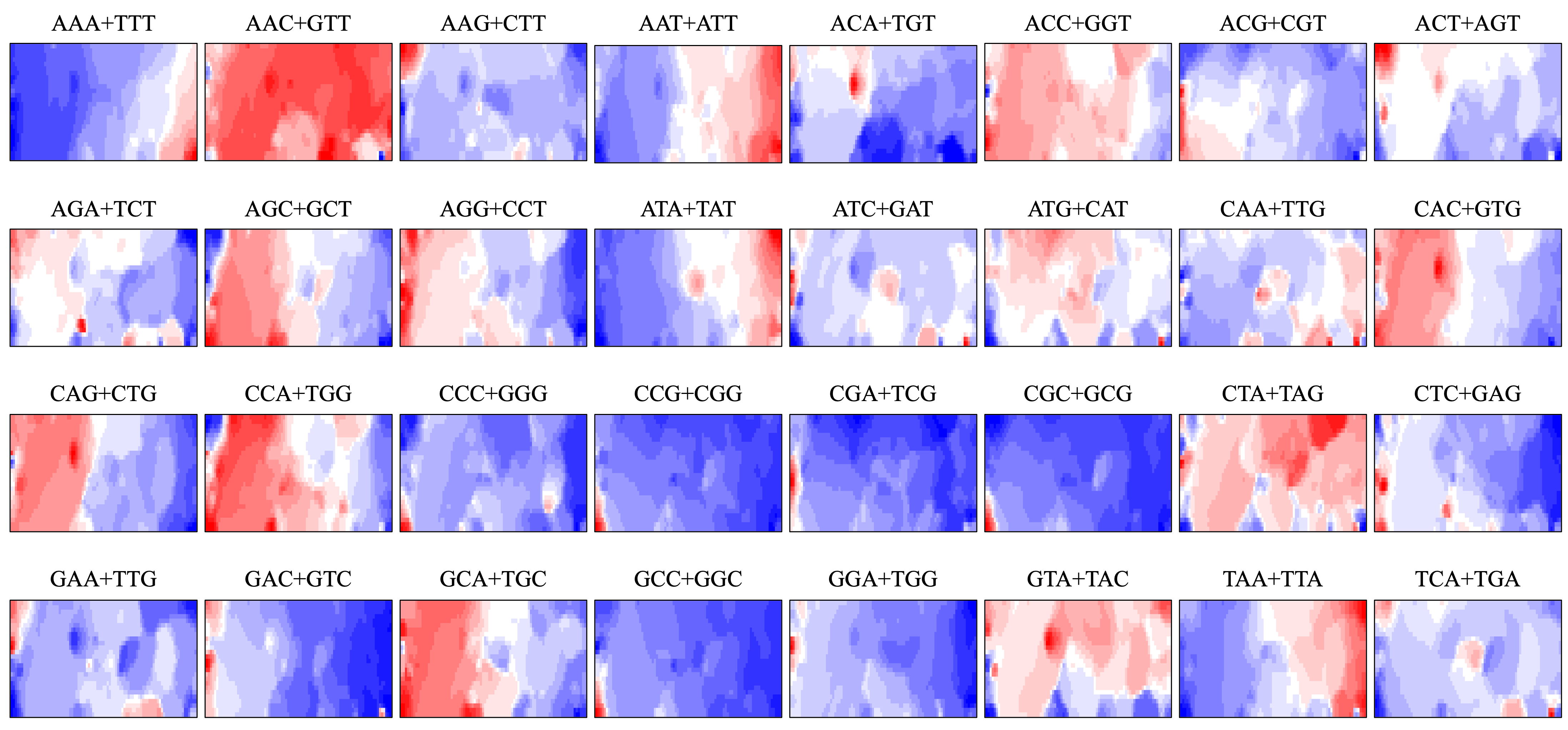

Supplement: Figure S10 — Contribution of each oligonucleotide at each node is visualized by color: dark red (very high), red (high), white (moderate), blue (low), and dark blue (very low). Orange/blue heatmap patterns were also presented, for the easy accessibility to those with non-normal color vision as Figure S19. [file peerj-12-17025-s010.png]

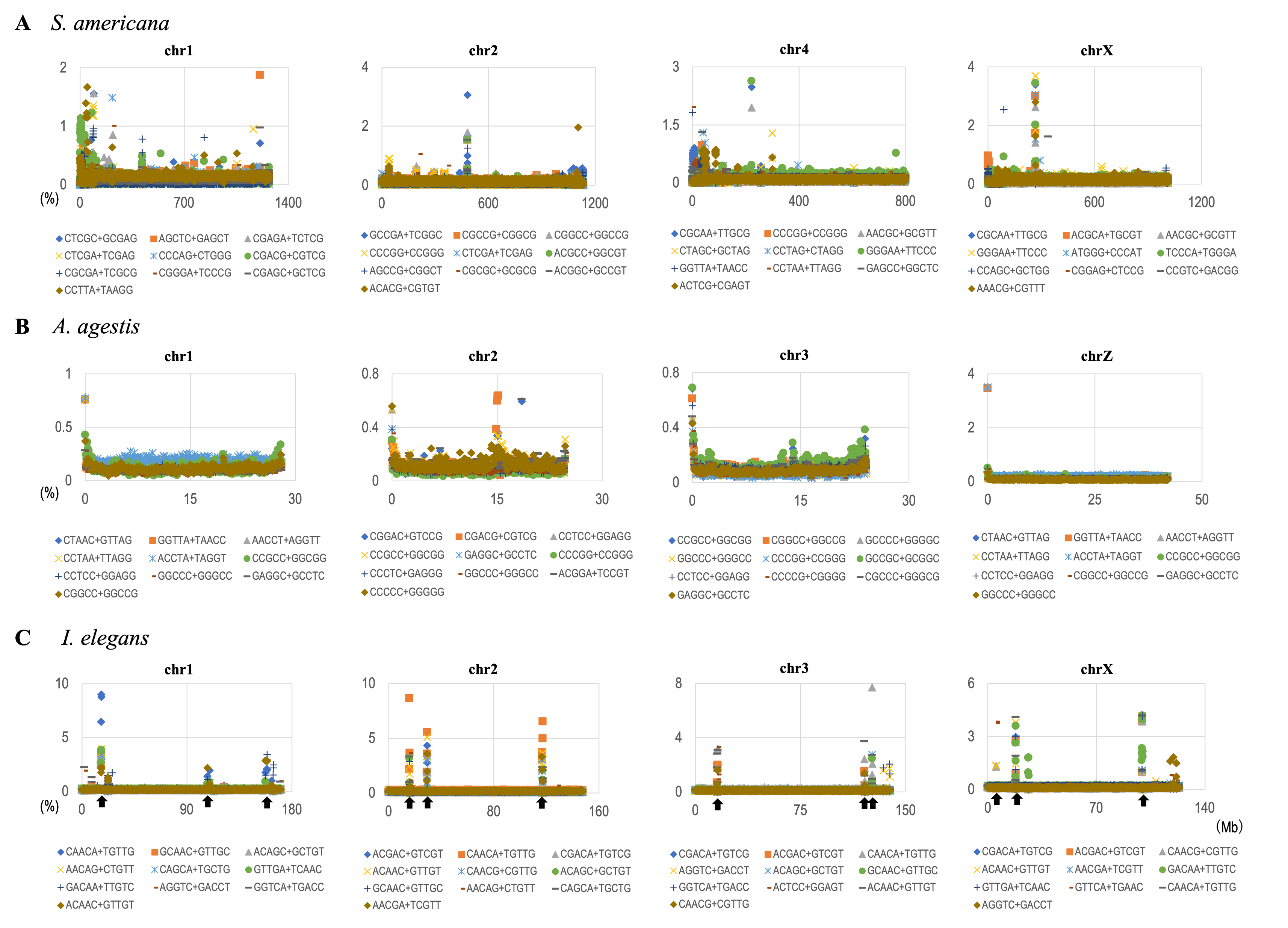

Supplement: Figure S11 — Distribution charts showing chromosomal distribution of DegePenta-nucleotide for (A) S. americana, (B) A. agestis, and (C) I. elegans are presented. The vertical axis represents the occurrence frequency (%) of each dinucleotide. [file peerj-12-17025-s011.png]

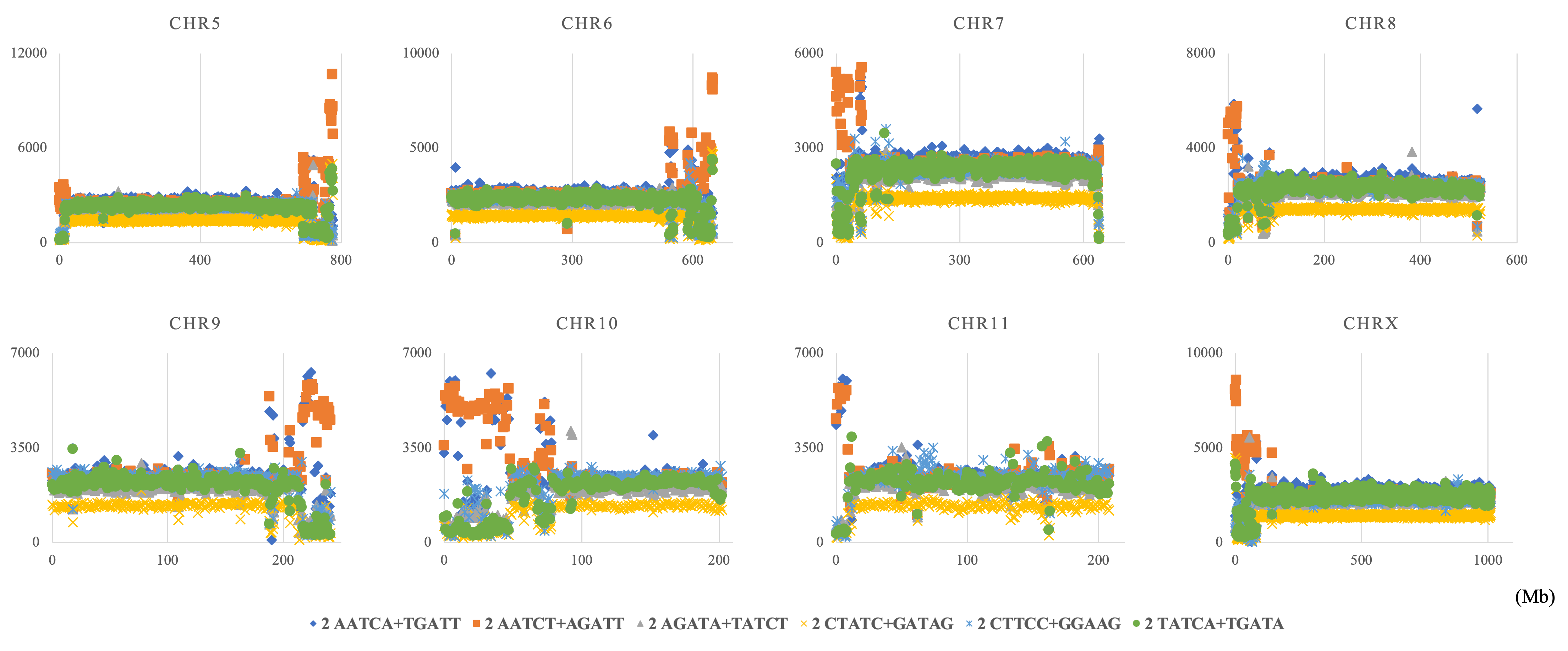

Supplement: Figure S12 — Distribution charts showing chromosomal distribution of six TFBS consensus core elements for S amerinaca are presented. The vertical axis represents the number of each TFBS consensus core element. [file peerj-12-17025-s012.png]

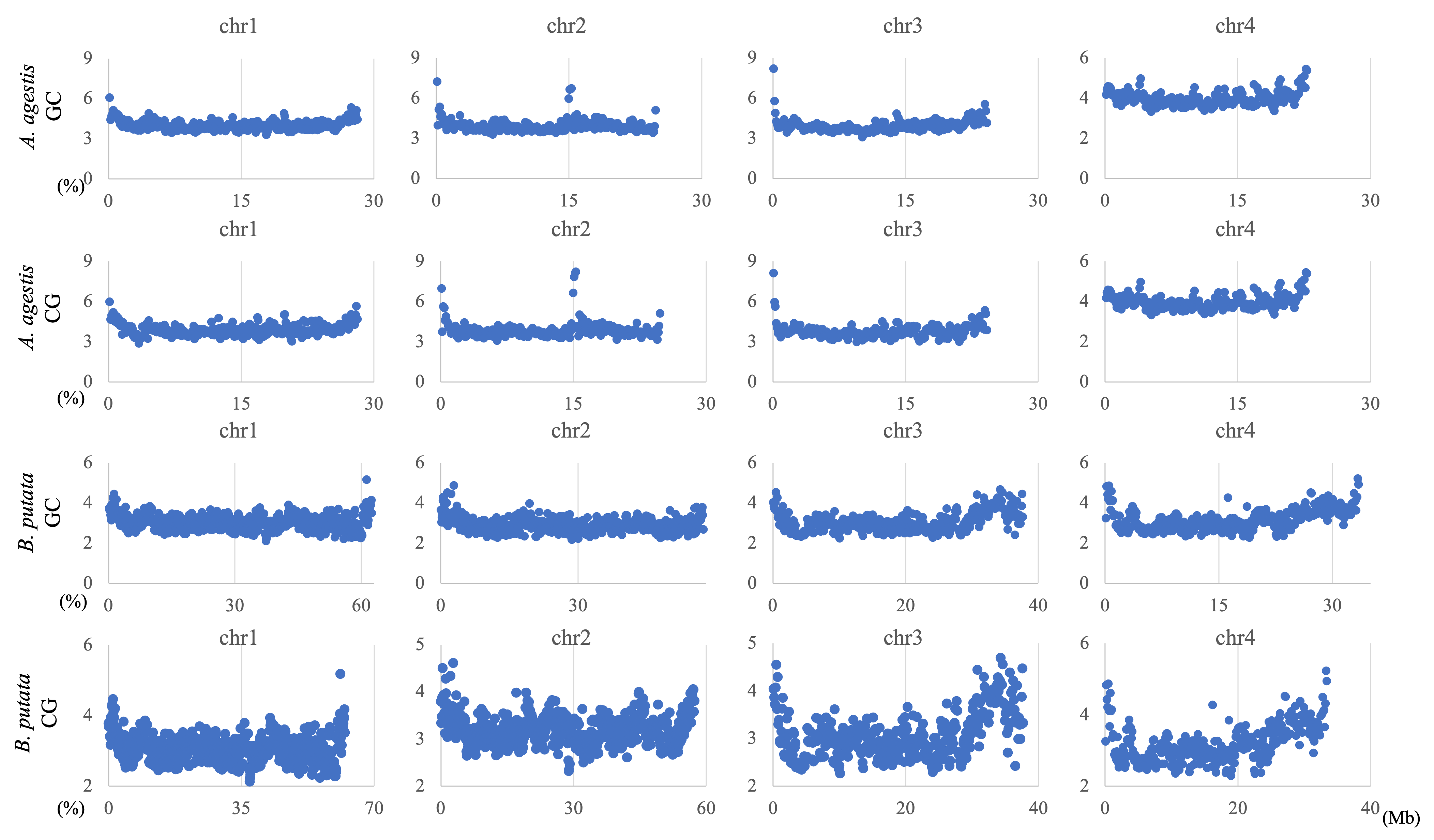

Supplement: Figure S13 — Distribution charts showing chromosomal distribution of CG and GC for A. agestis and and B. putata on four chromosomes are presented. The vertical axis represents the occurrence frequency (%) of each DegeDi. [file peerj-12-17025-s013.png]

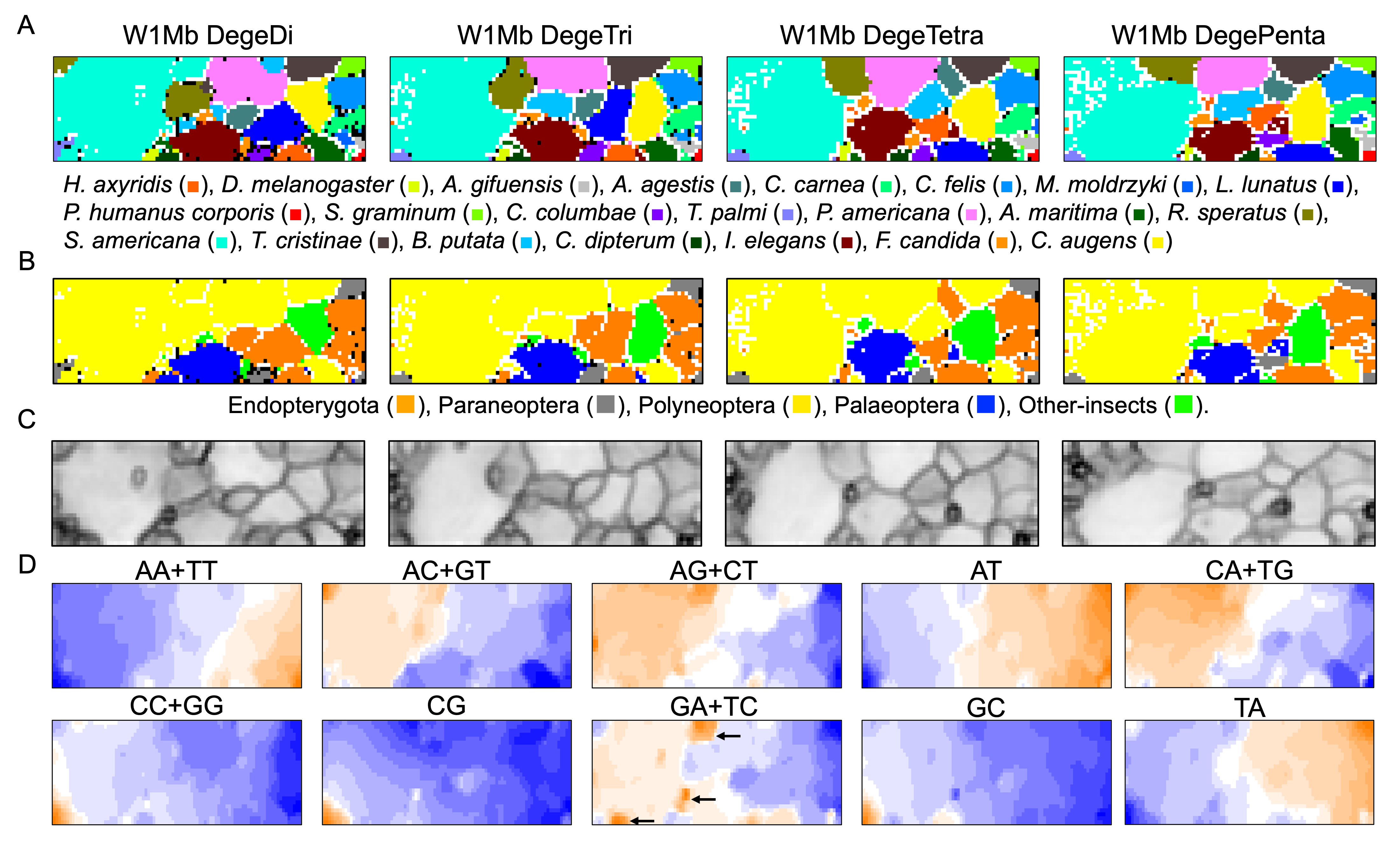

Supplement: Figure S14 — Another version of the heatmap in Figure 1, where the red/blue heatmap pattern has been changed to an orange/blue heatmap pattern for the easy accessibility to those with non-normal color vision. [file peerj-12-17025-s014.png]

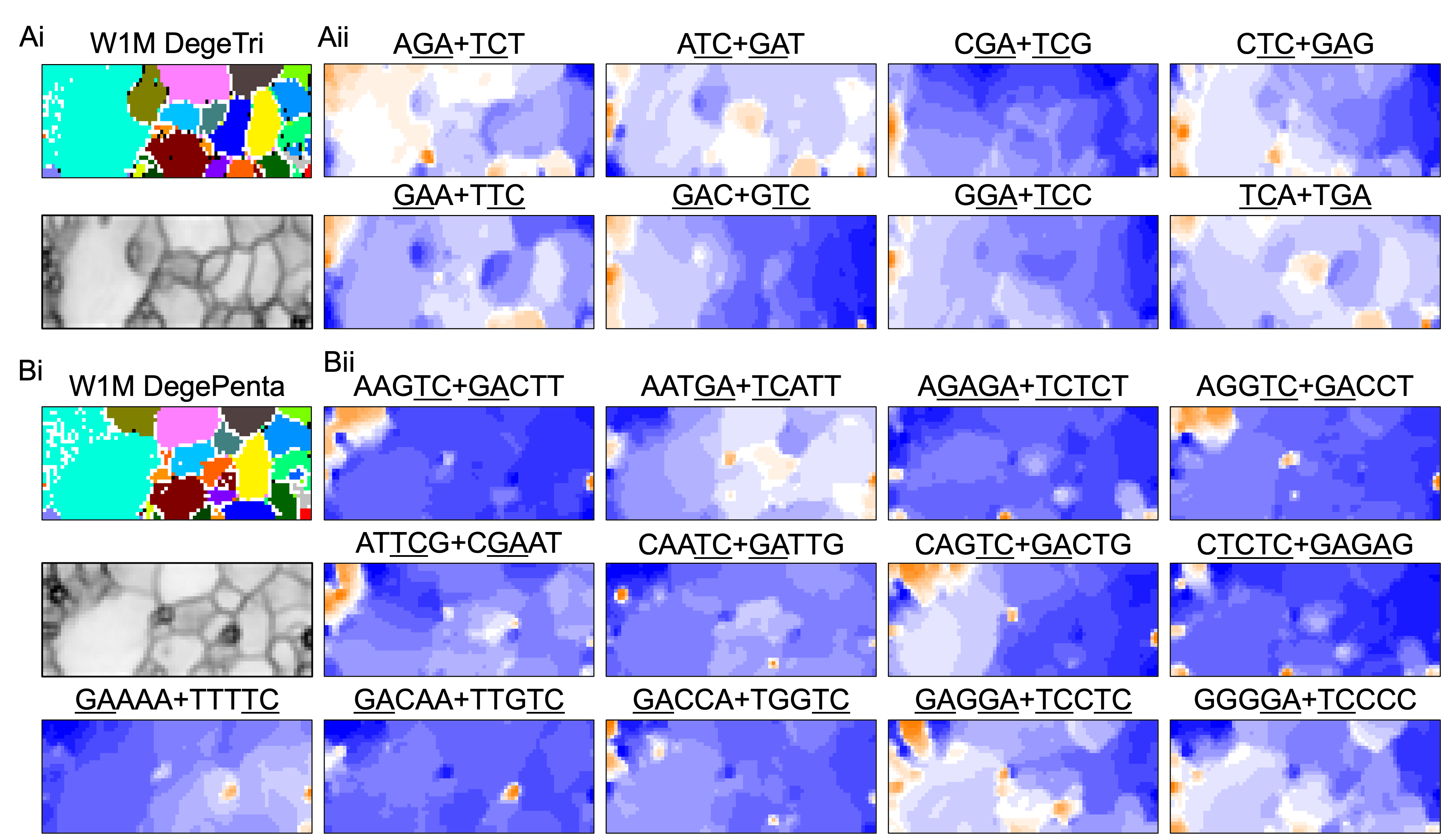

Supplement: Figure S15 — Another version of the heatmap in Figure 5, where the red/blue heatmap pattern has been changed to an orange/blue heatmap pattern for the easy accessibility to those with non-normal color vision. [file peerj-12-17025-s015.png]

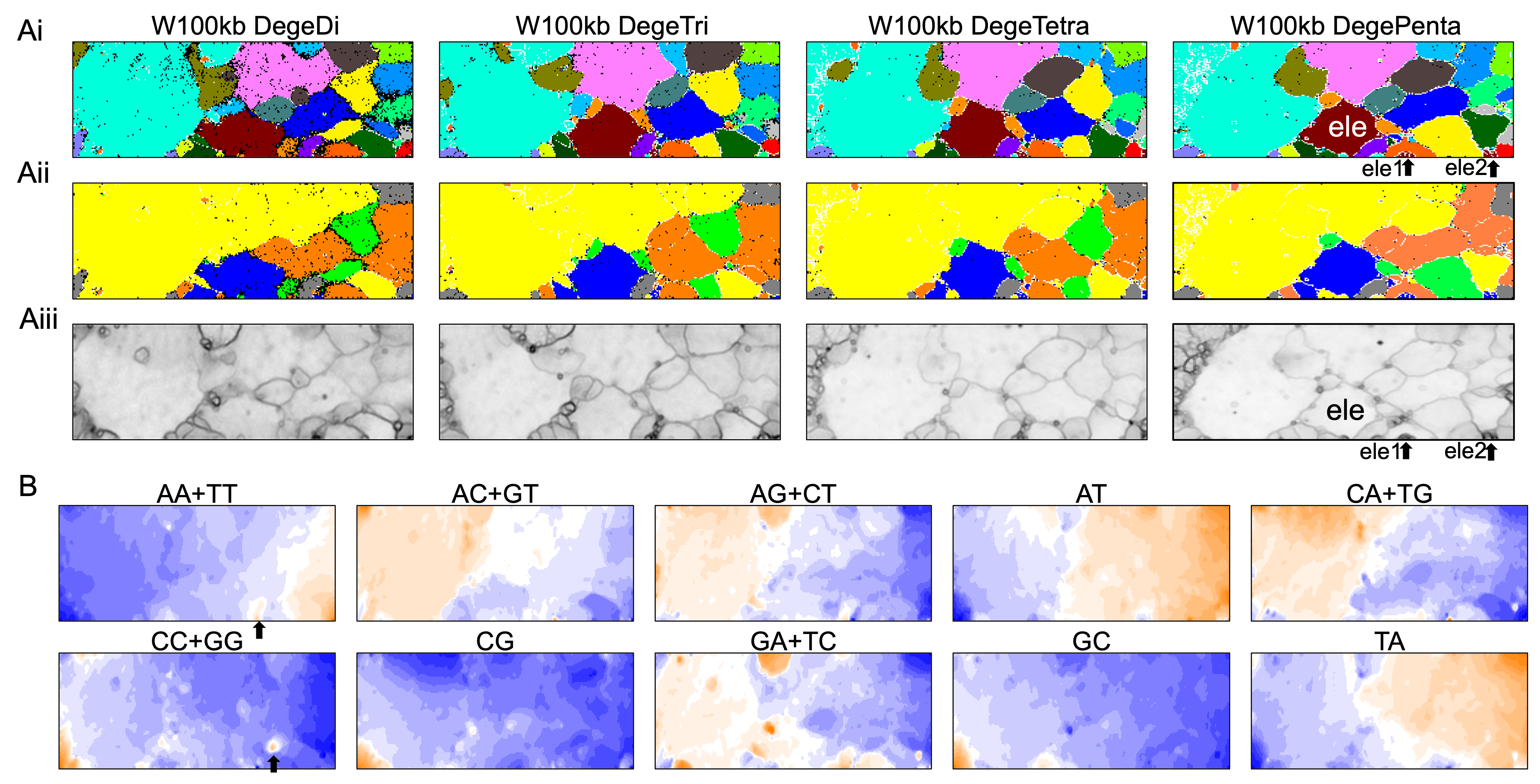

Supplement: Figure S16 — Another version of the heatmap in Figure 6, where the red/blue heatmap pattern has been changed to an orange/blue heatmap pattern for the easy accessibility to those with non-normal color vision. [file peerj-12-17025-s016.png]

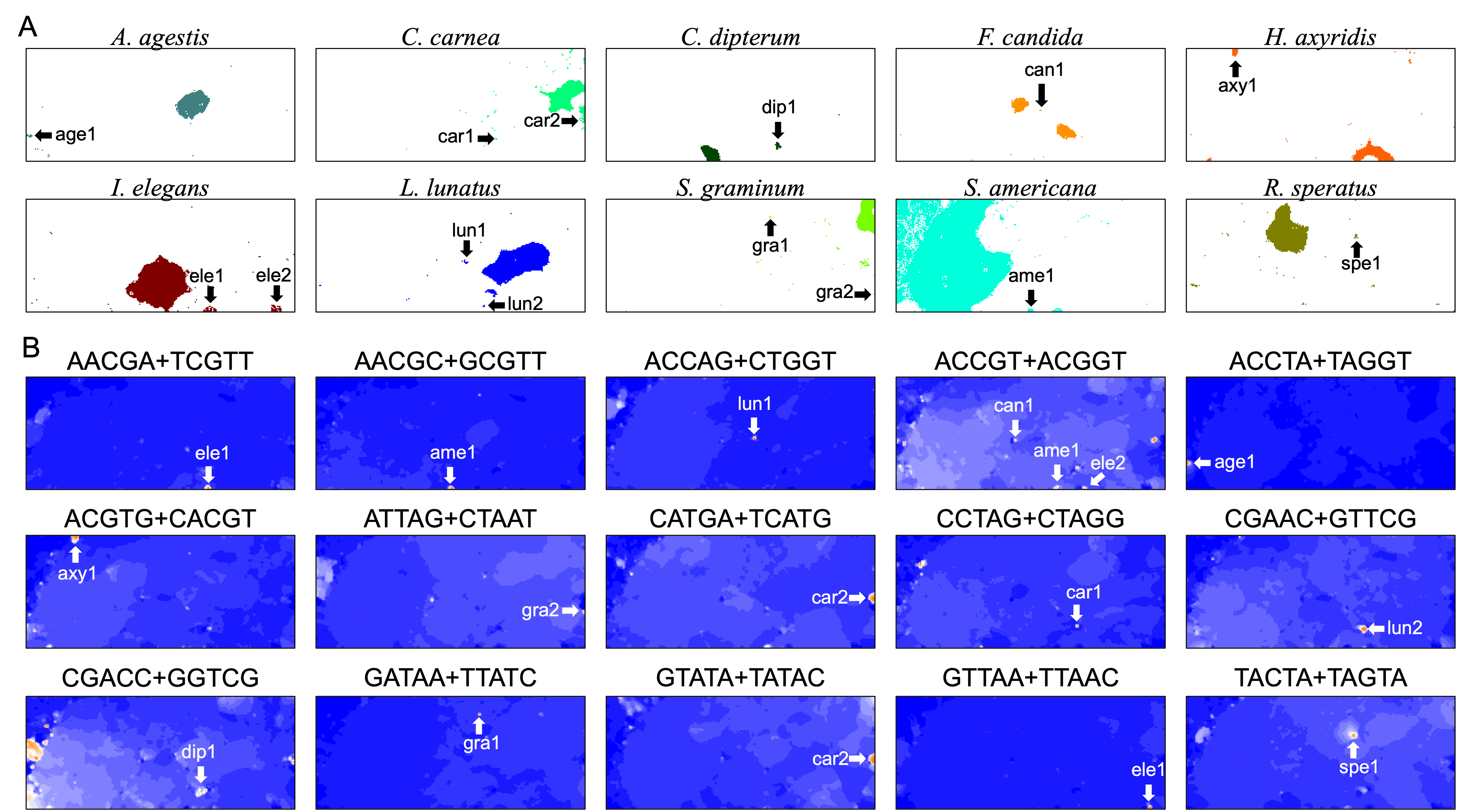

Supplement: Figure S17 — Another version of the heatmap in Figure 8, where the red/blue heatmap pattern has been changed to an orange/blue heatmap pattern for the easy accessibility to those with non-normal color vision. [file peerj-12-17025-s017.png]

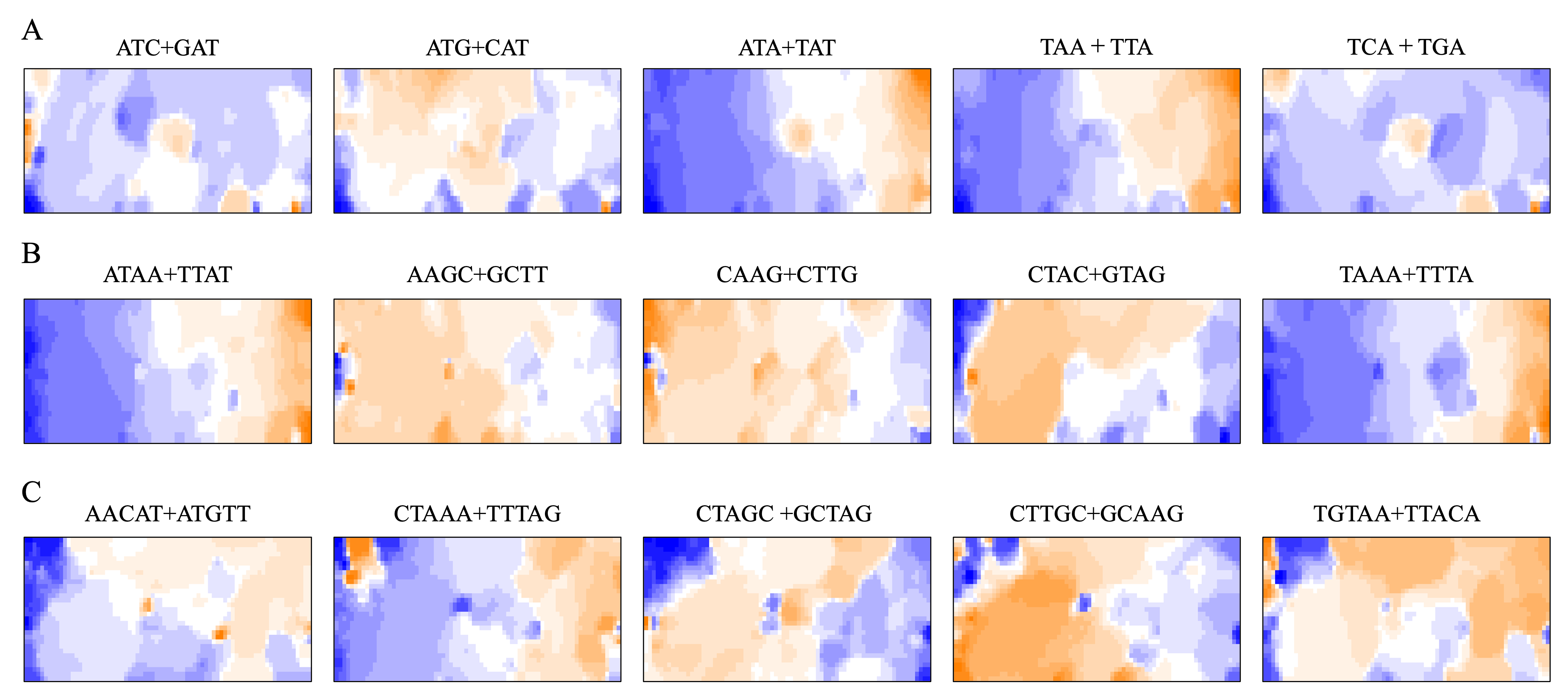

Supplement: Figure S18 — Another version of the heatmap in Figure S6, where the red/blue heatmap pattern has been changed to an orange/blue heatmap pattern for the easy accessibility to those with non-normal color vision. [file peerj-12-17025-s018.png]

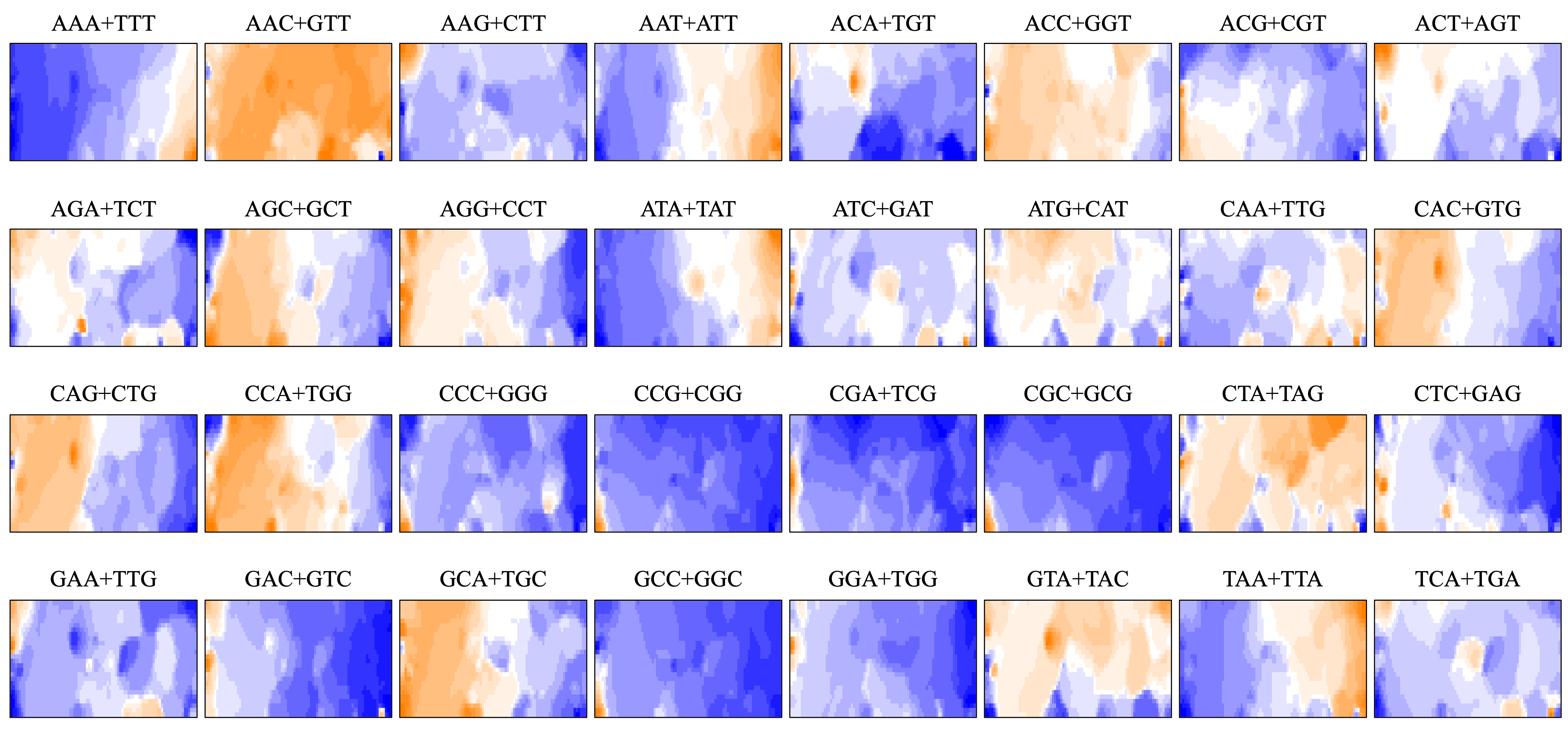

Supplement: Figure S19 — Another version of the heatmap in Figure S19, where the red/blue heatmap pattern has been changed to an orange/blue heatmap pattern for the easy accessibility to those with non-normal color vision. [file peerj-12-17025-s019.png]

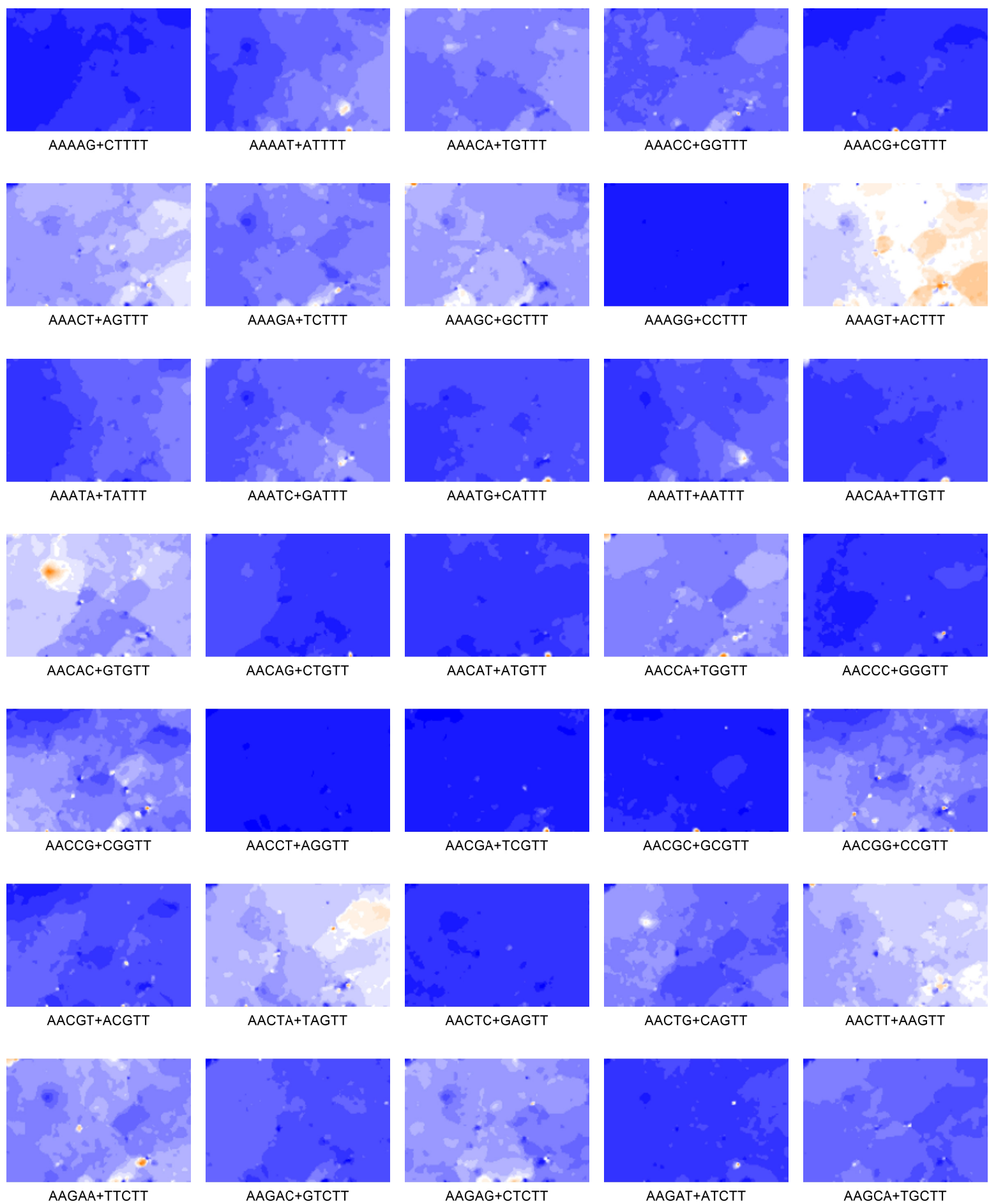

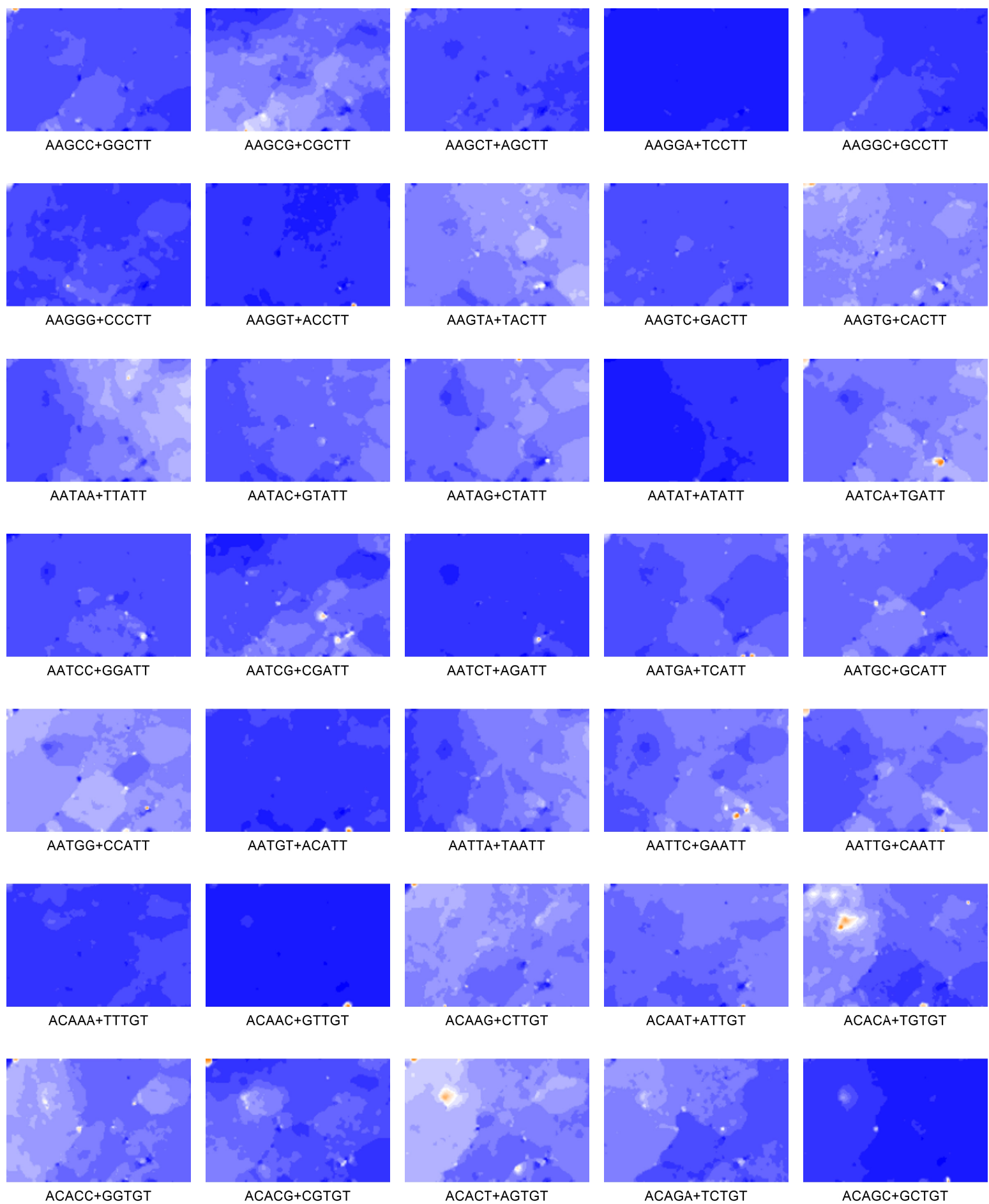

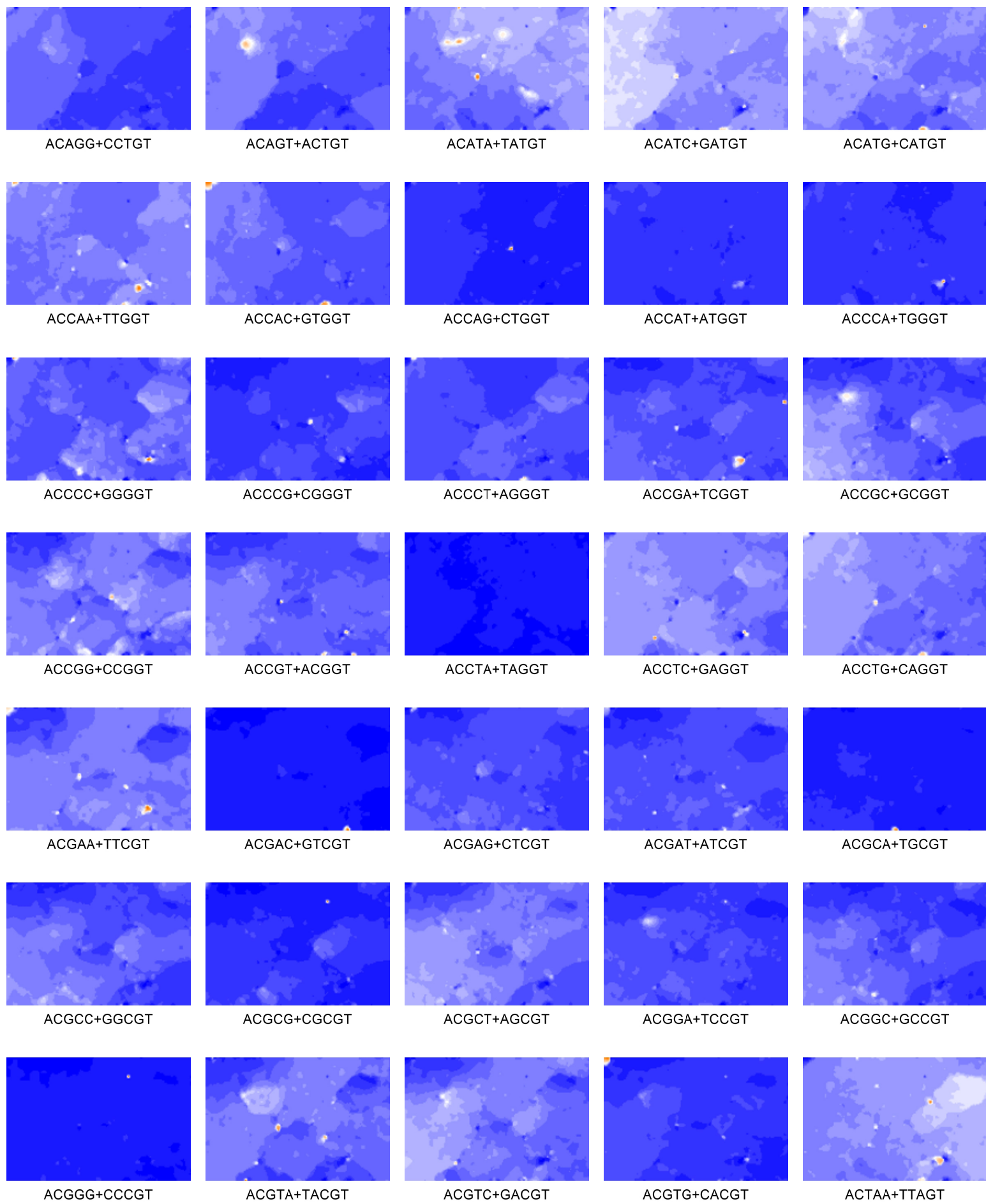

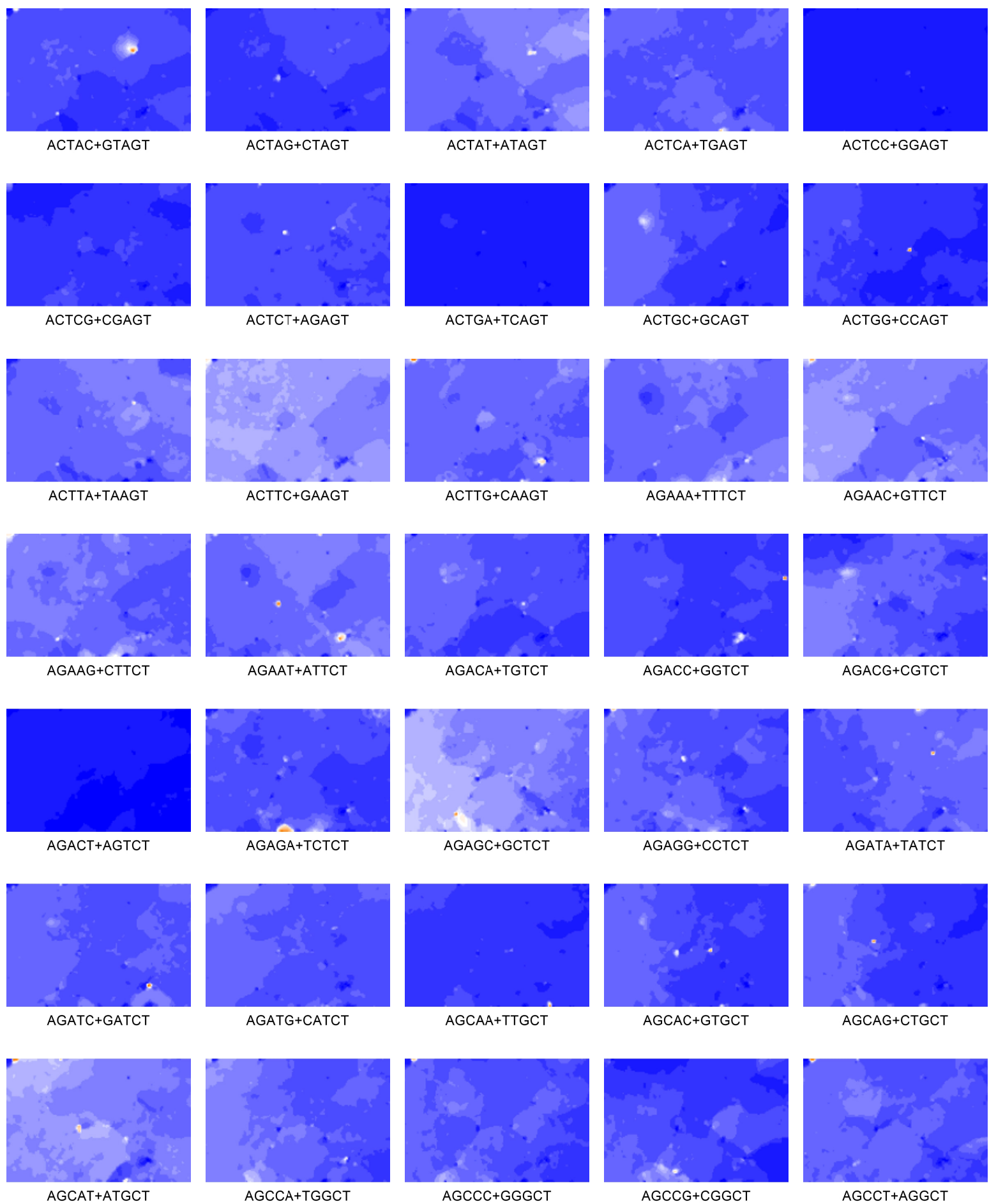

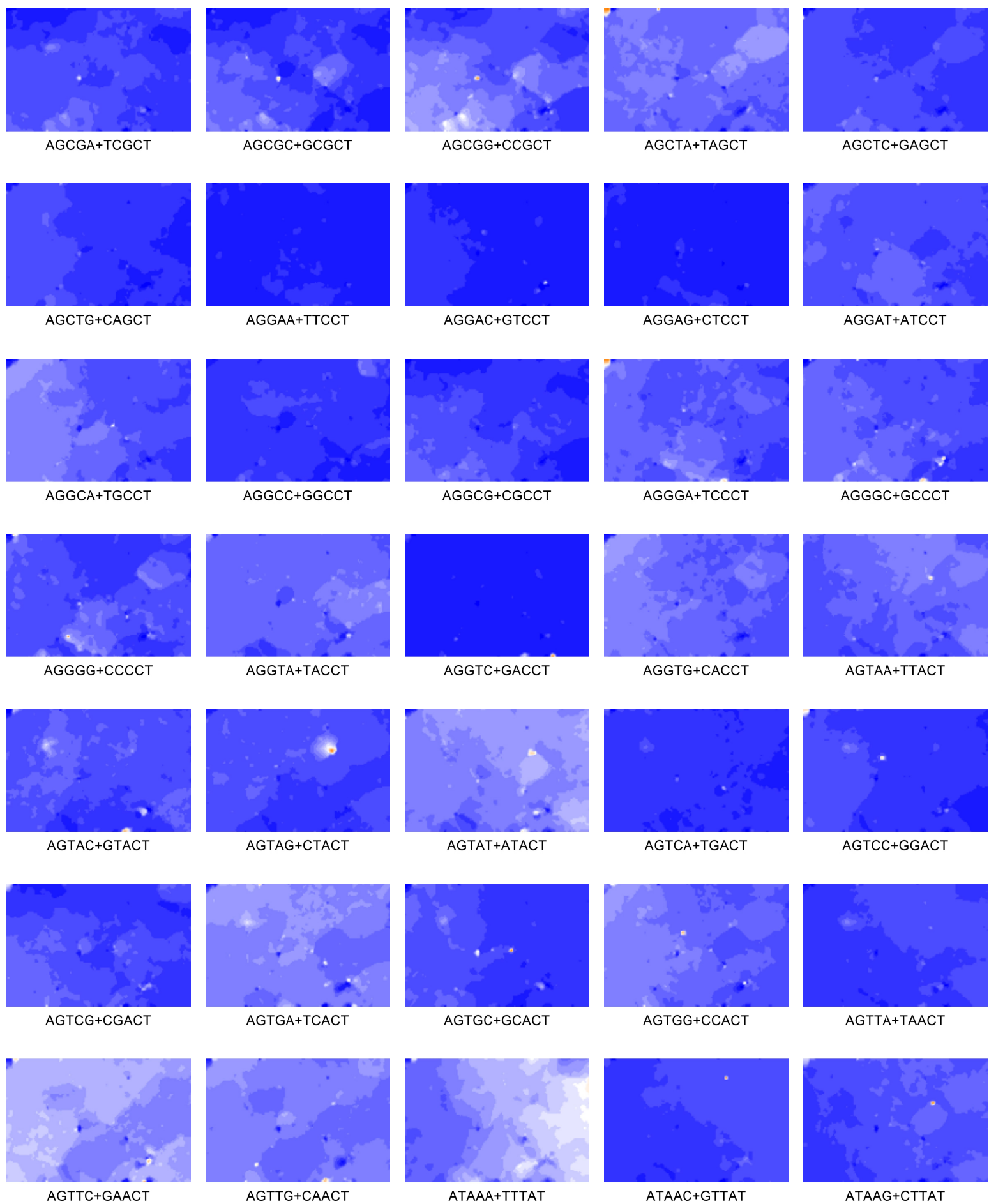

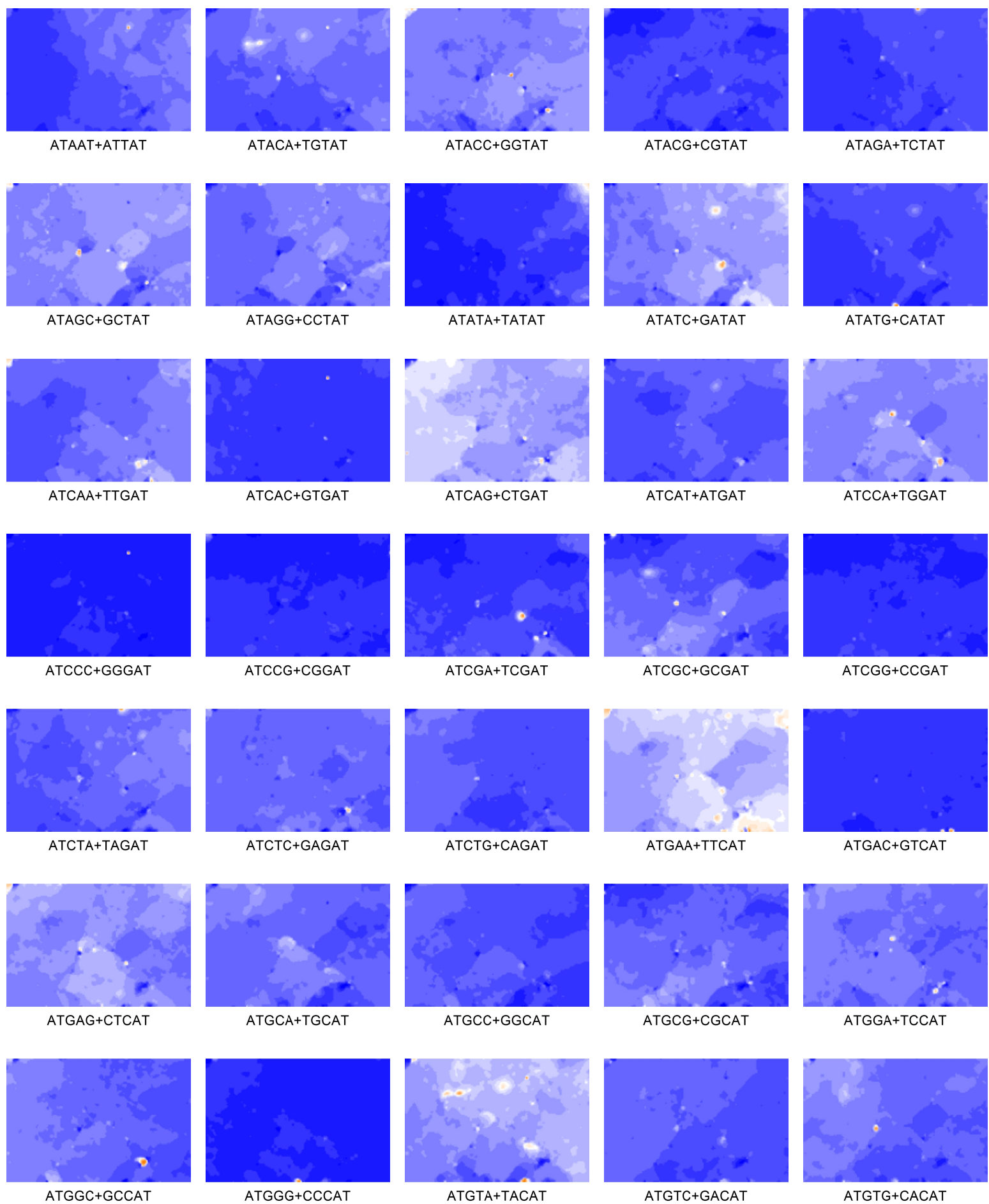

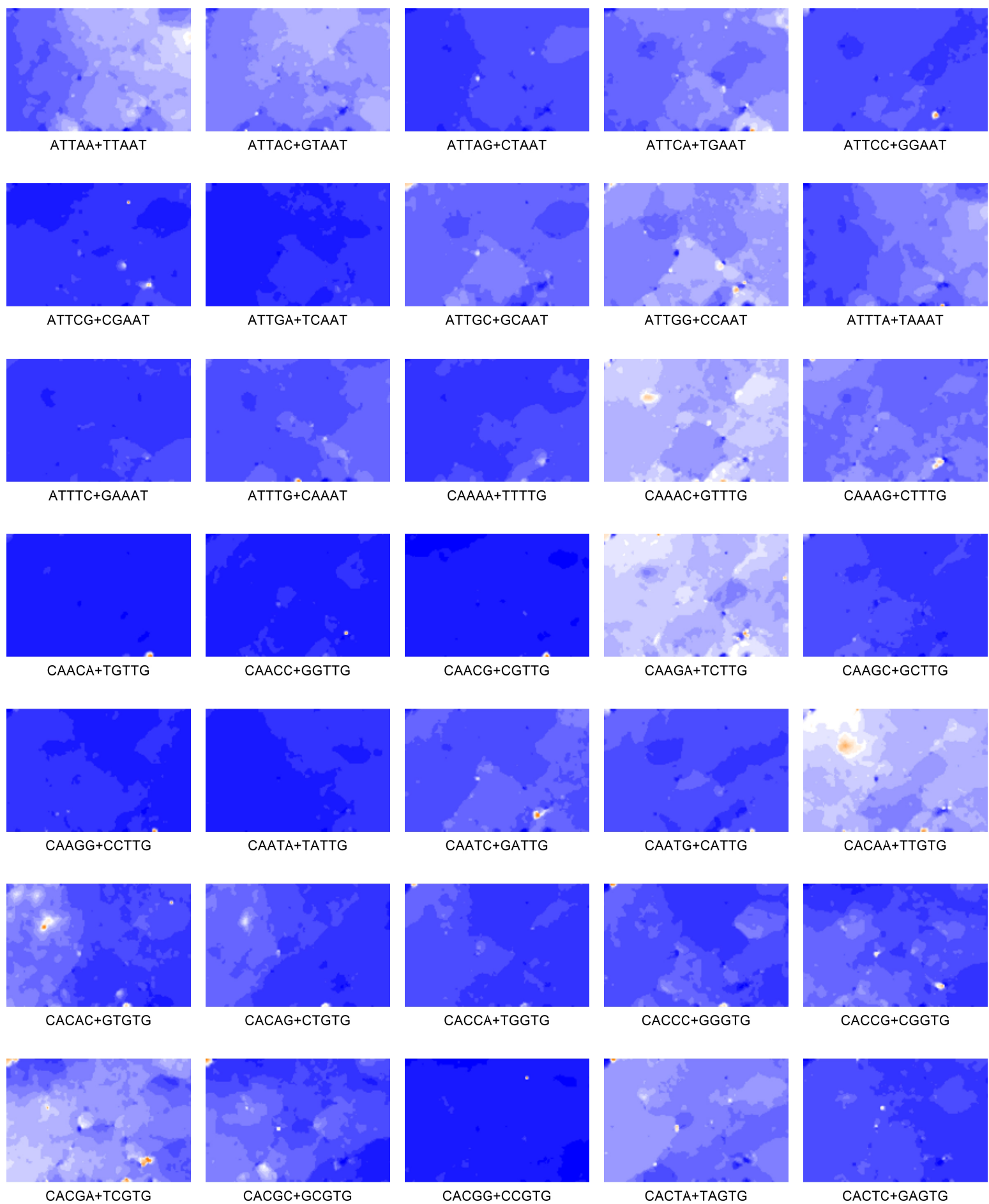

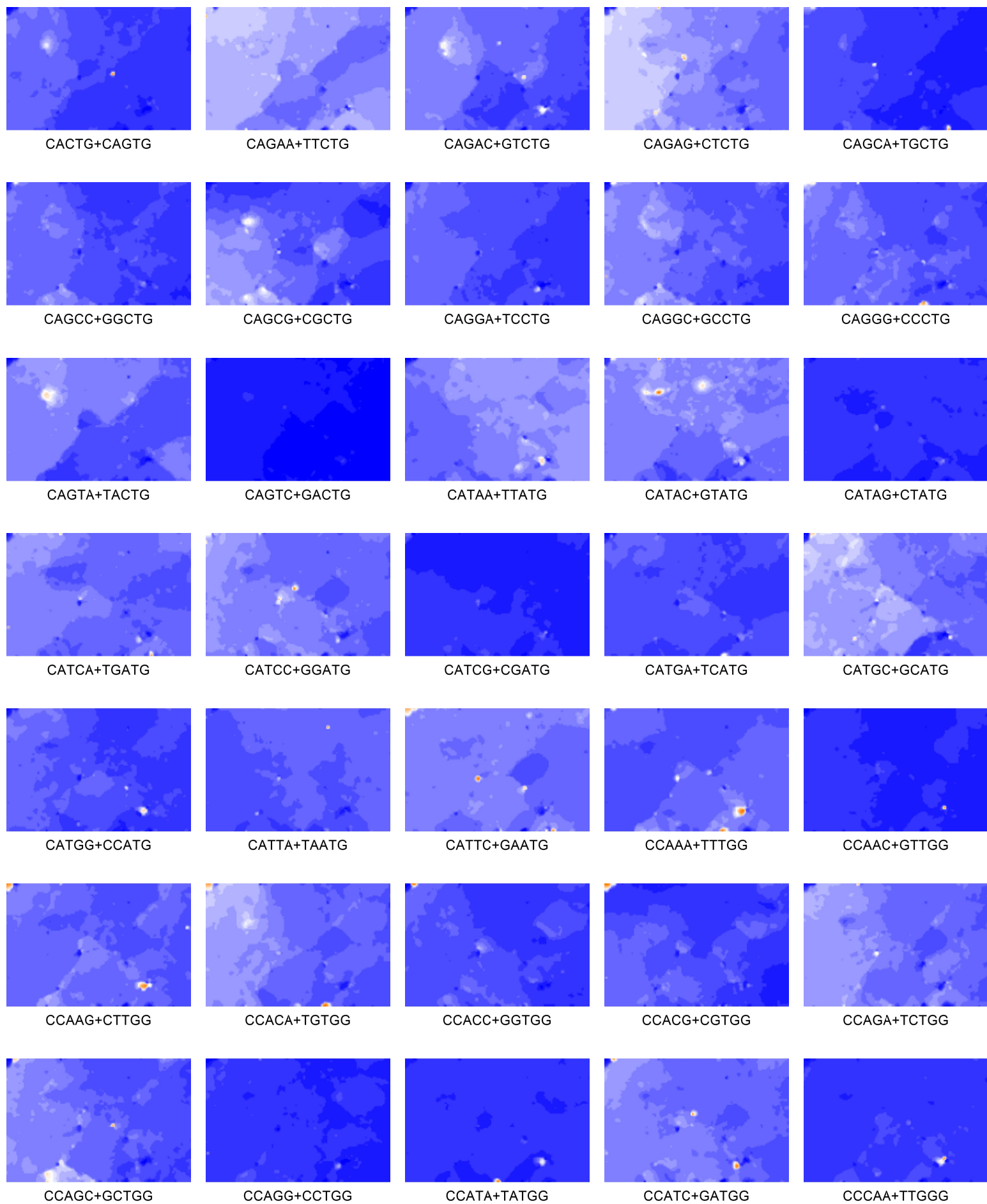

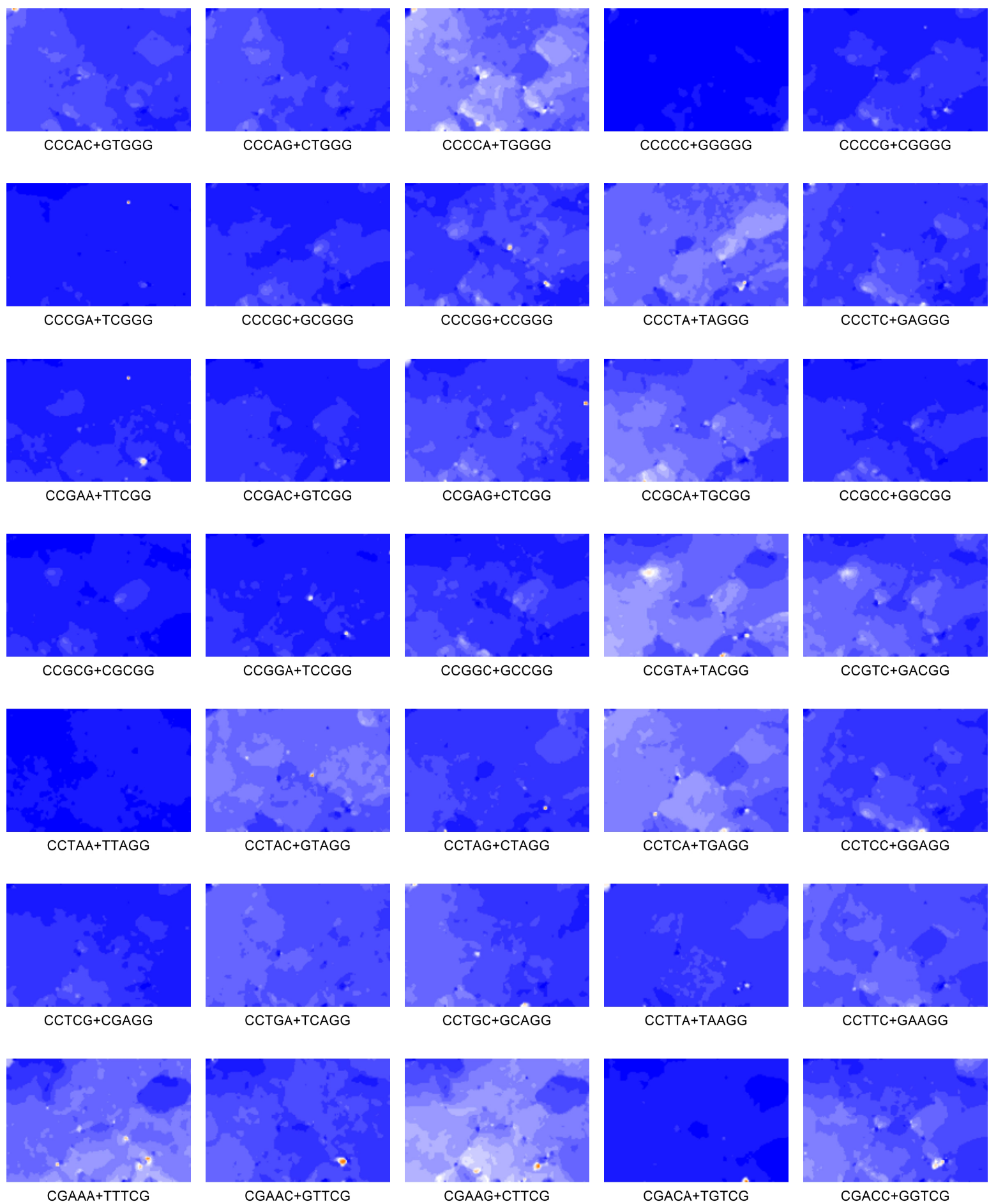

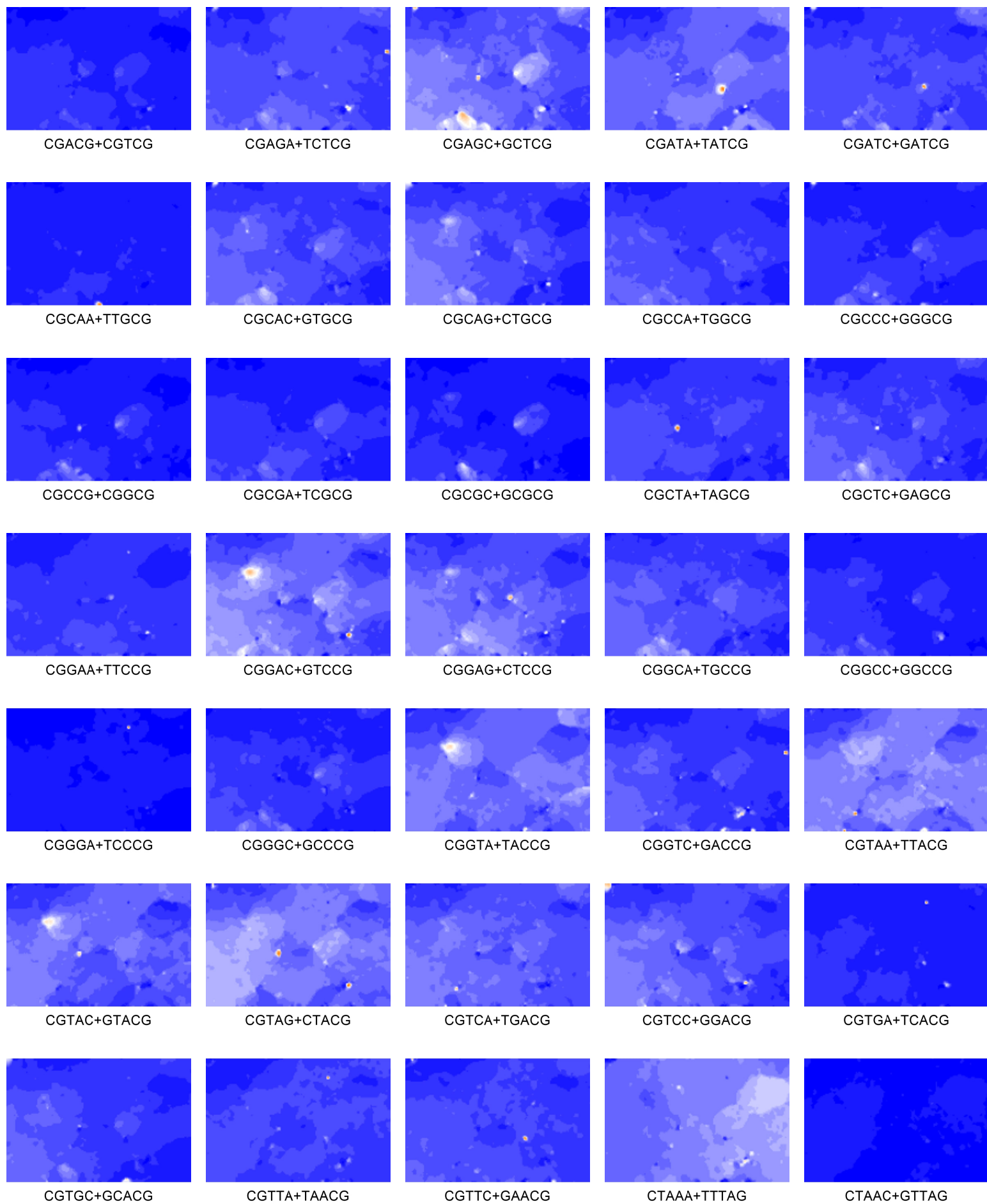

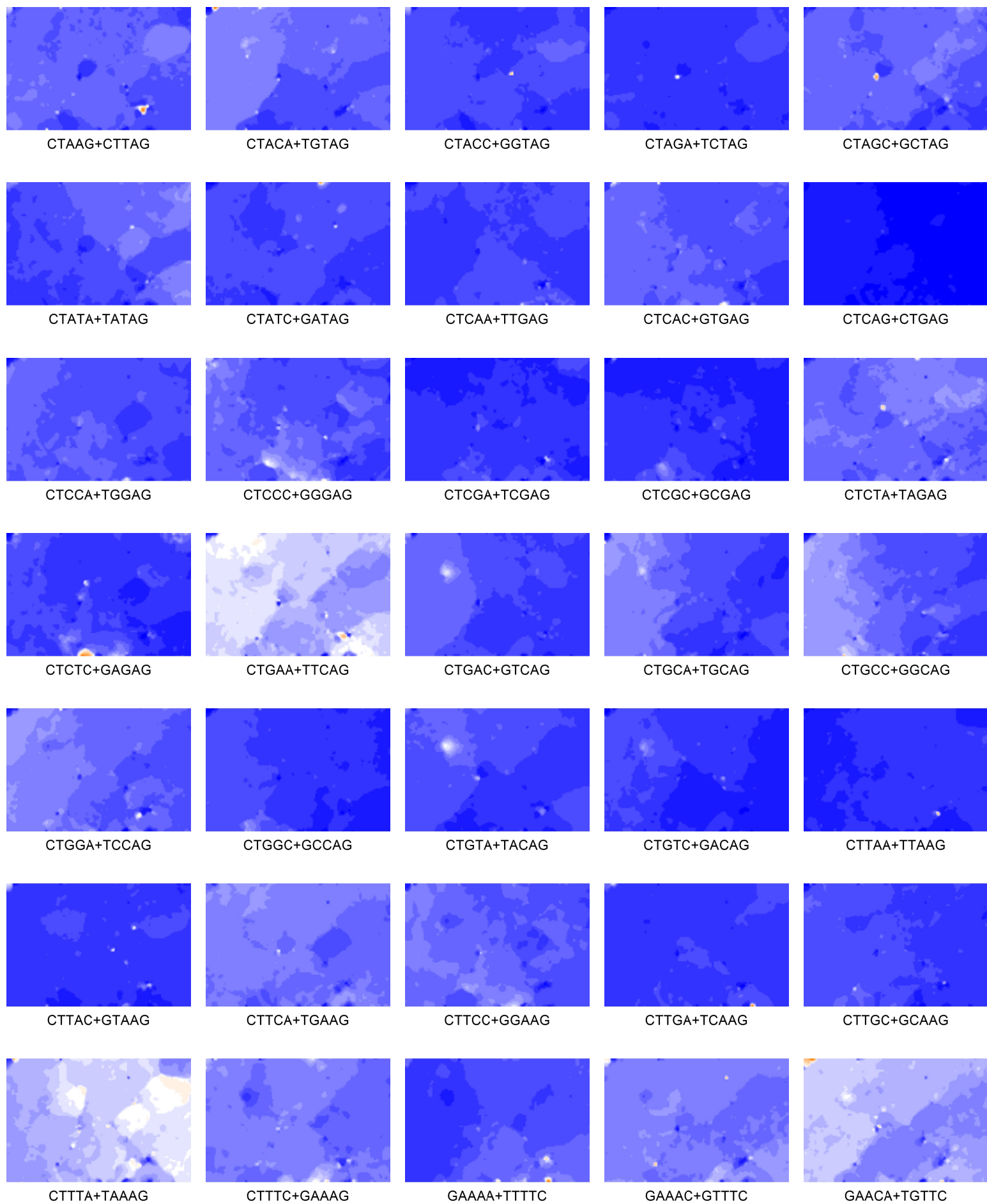

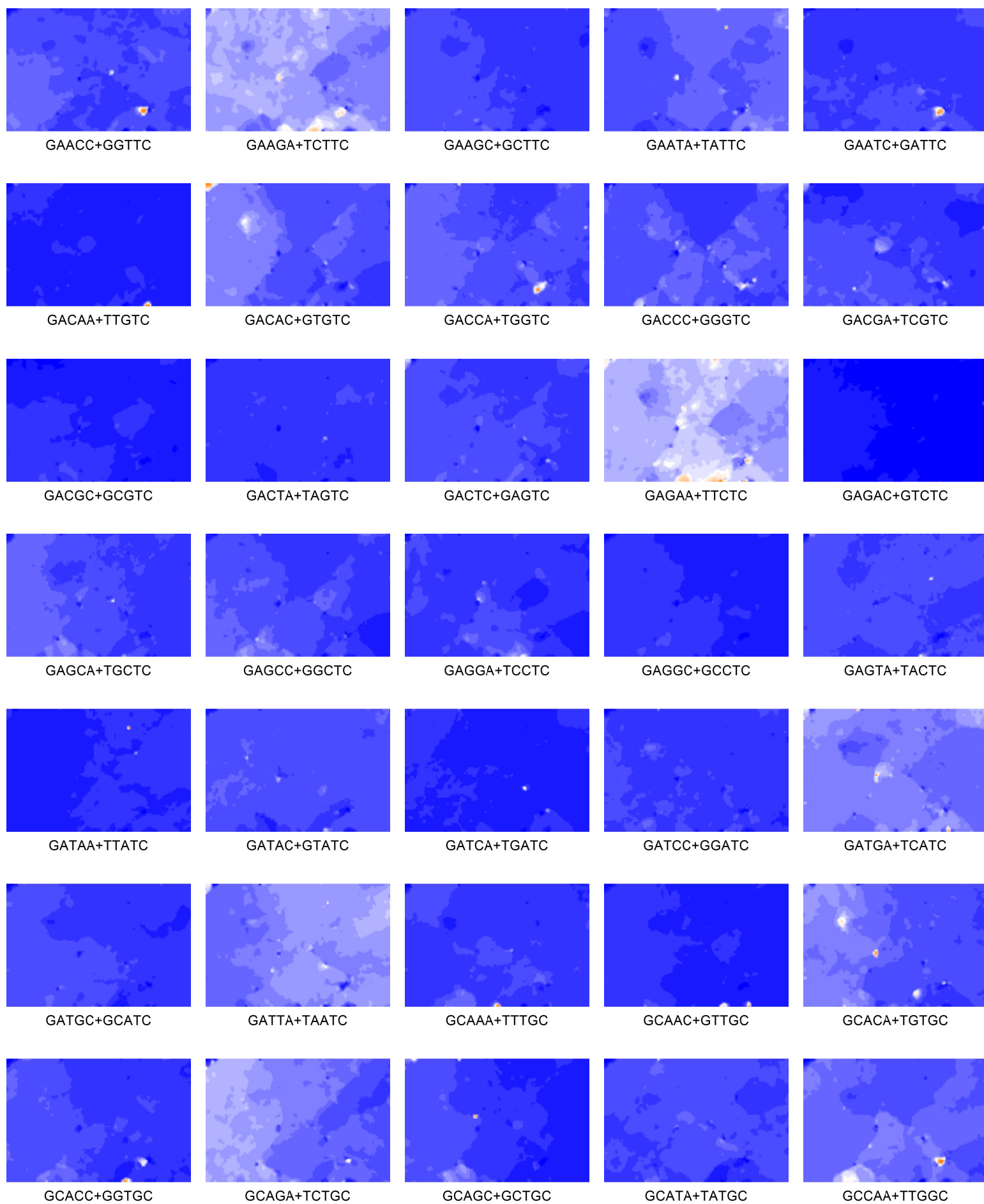

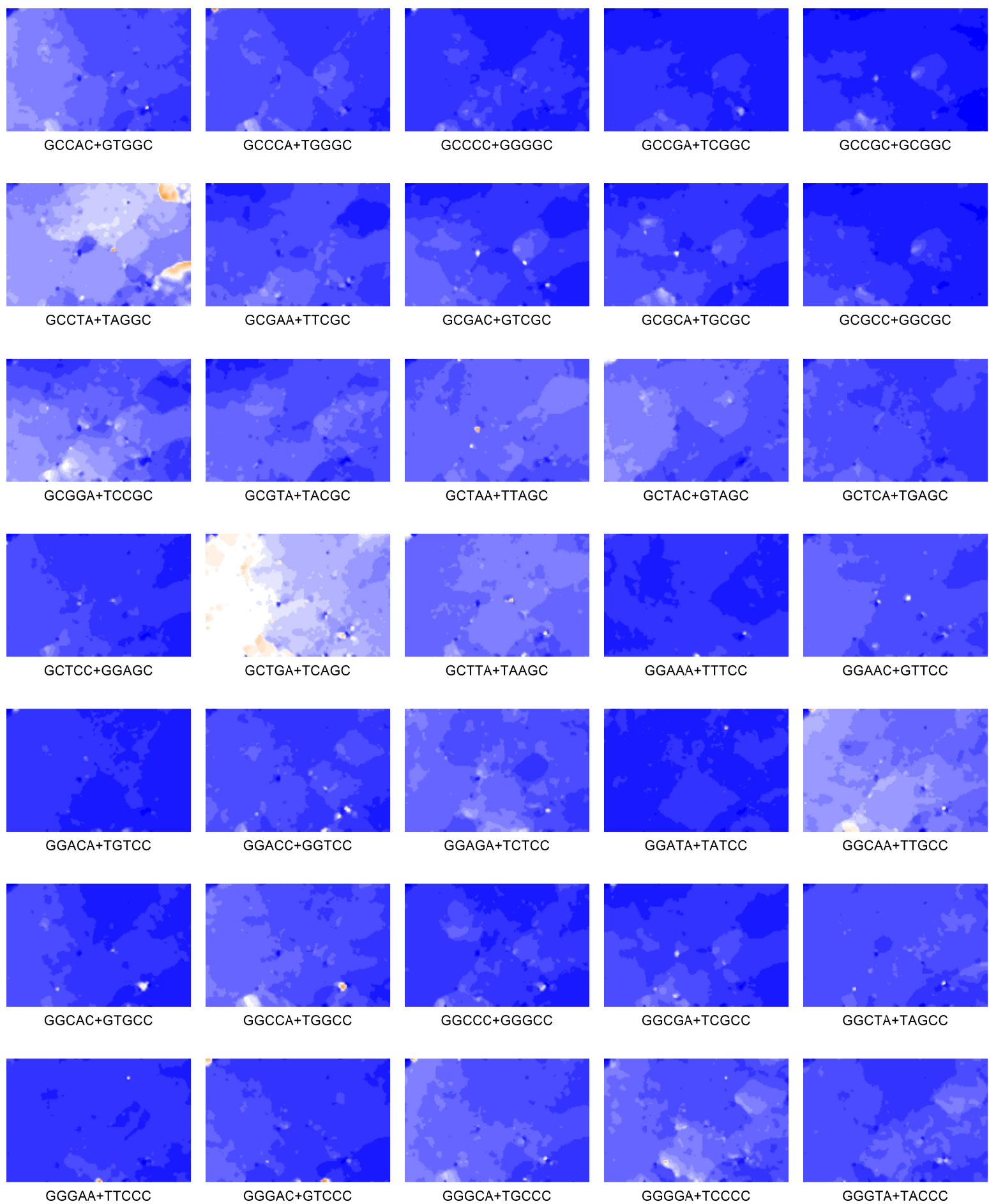

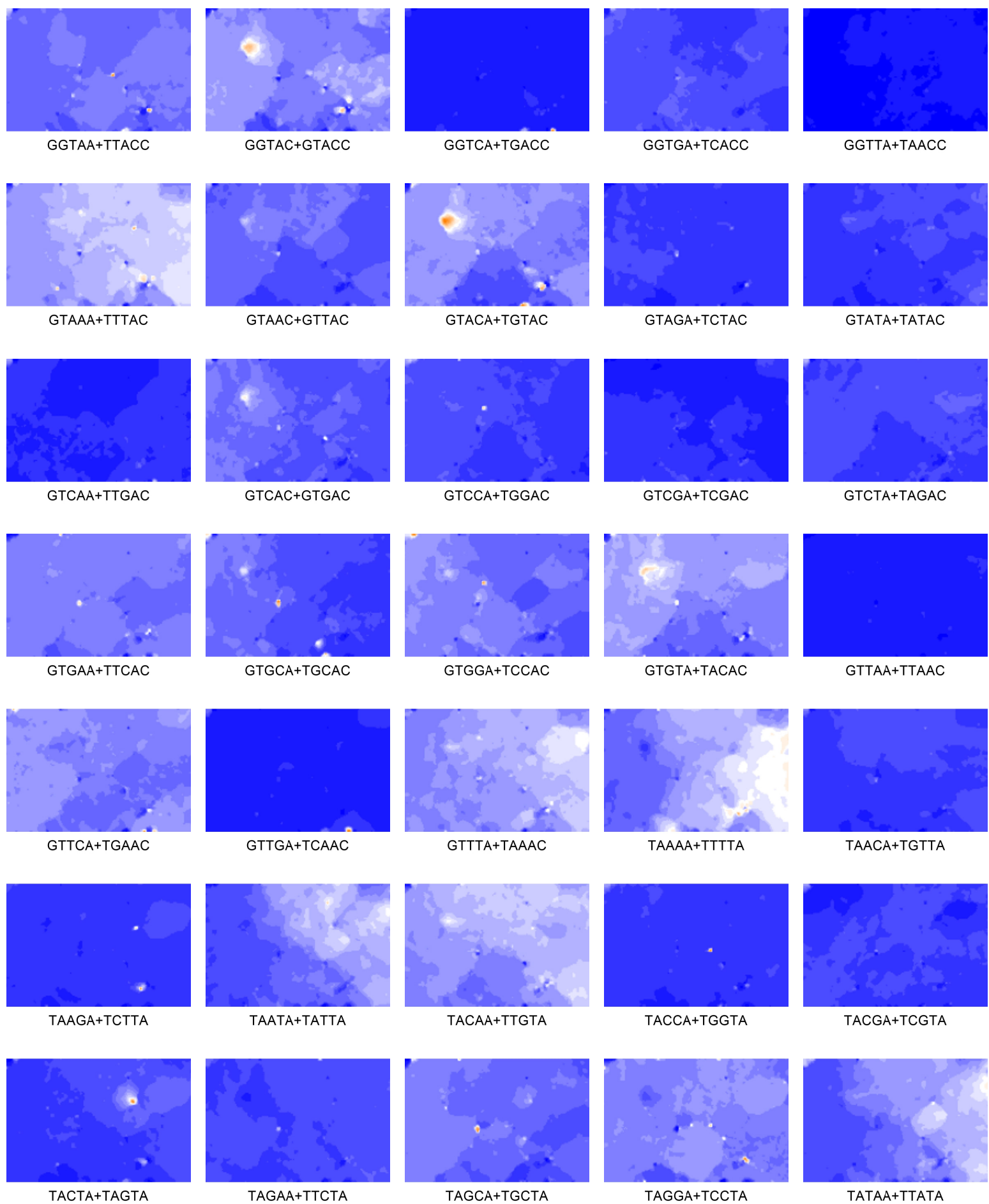

Supplement: Data S4 — Another version of the heatmap in Data S2, where the red/blue heatmap pattern has been changed to an orange/blue heatmap pattern for the easy accessibility to those with non-normal color vision. [file peerj-12-17025-s028.pdf]
