# Supplementary material for: Unsupervised AI reveals insect species-specific genome signatures
Source: PeerJ. 2024 Mar 6;12:e17025. doi: 10.7717/peerj.17025 (PMC10924456; doi:10.7717/peerj.17025)
Supplement: Data S1 — Orange/blue heatmap patterns were also presented, for the easy accessibility to those with non-normal color vision as Data S3. [file peerj-12-17025-s025.pdf]

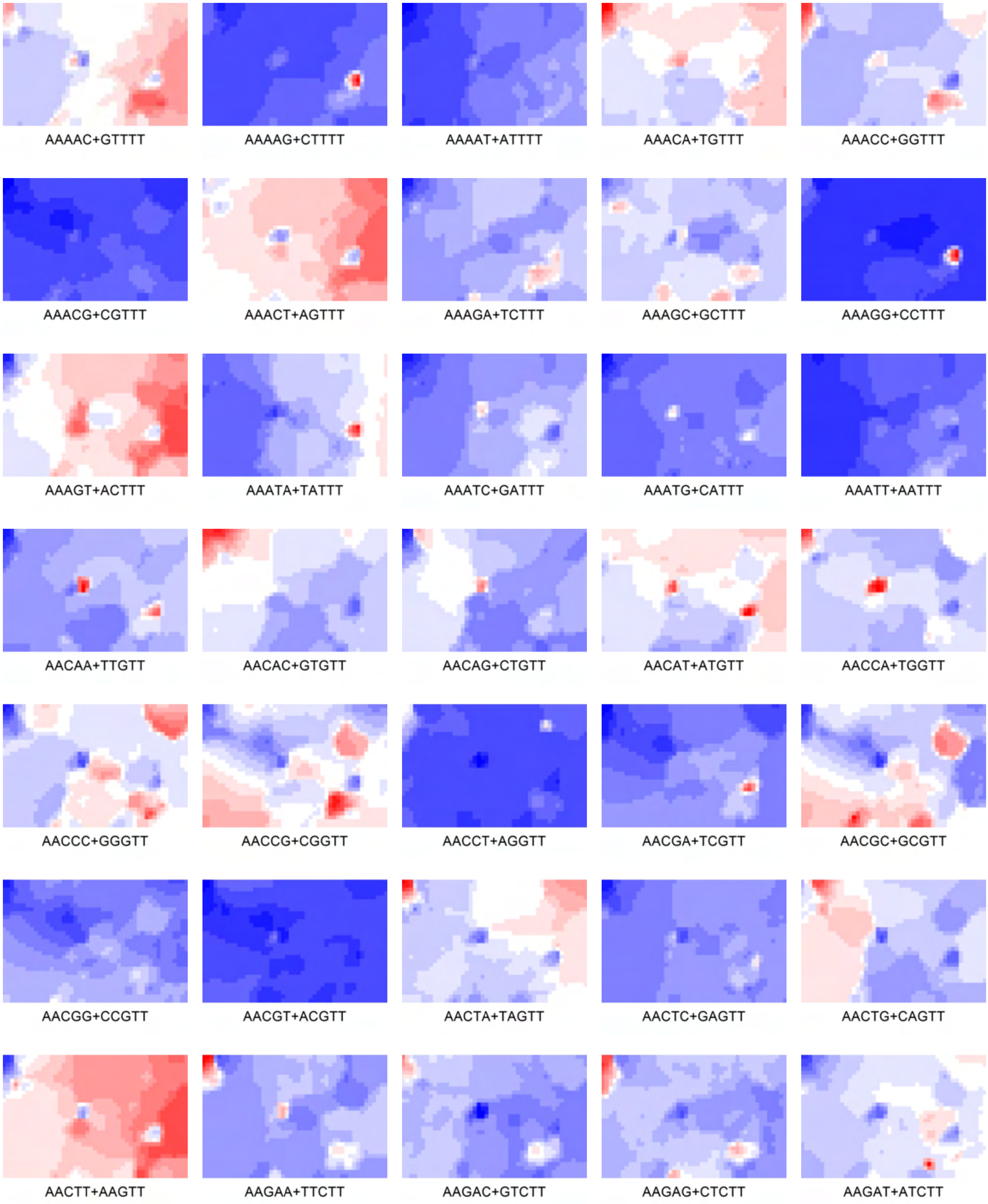
AAAAC+GTTTT
AAAAG+CTTTT
AAAAT+ATTTT
AAACA+TGTTT
AAACC+GGTTT
AAACG+CGTTT
AAACT+AGTTT
AAAGA+TCTTT
AAAGC+GCTTT
AAAGG+CCTTT
AAAGT+ACTTT
AAATA+TATTT
AAATC+GATTT
AAATG+CATTT
AAATT+AATTT
AACAA+TTGTT
AACAC+GTGTT
AACAG+CTGTT
AACAT+ATGTT
AACCA+TGGTT
AACCC+GGGTT
AACCG+CGGTT
AACCT+AGGTT
AACGA+TCGTT
AACGC+GCGTT
AACGG+CCGTT
AACGT+ACGTT
AACTA+TAGTT
AACTC+GAGTT
AACTG+CAGTT
AACTT+AAGTT
AAGAA+TTCTT
AAGAC+GTCTT
AAGAG+CTCTT
AAGAT+ATCTT

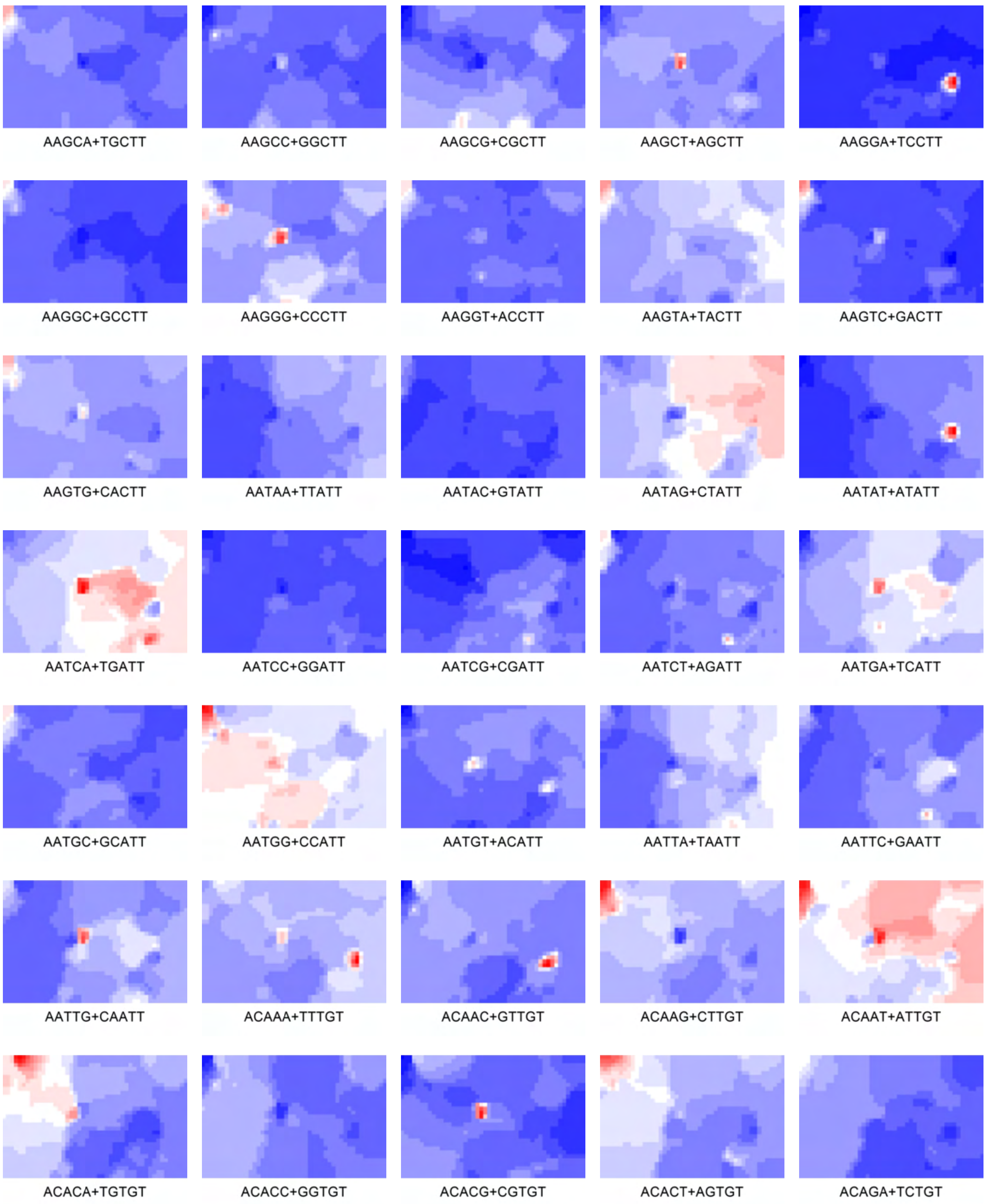
AAGCA+TGCTT
AAGCC+GGCTT
AAGCG+CGCTT
AAGCT+AGCTT
AAGGA+TCCTT
AAGGC+GCCTT
AAGGG+CCCTT
AAGGT+ACCTT
AAGTA+TACTT
AAGTC+GACTT
AAGTG+CACTT
AATAA+TTATT
AATAC+GTATT
AATAG+CTATT
AATAT+ATATT
AATCA+TGATT
AATCC+GGATT
AATCG+CGATT
AATCT+AGATT
AATGA+TCATT
AATGC+GCATT
AATGG+CCATT
AATGT+ACATT
AATTA+TAATT
AATTC+GAATT
AATTG+CAATT
ACAAA+TTTGT
ACAAC+GTTGT
ACAAG+CTTGT
ACAAT+ATTGT
ACACA+TGTGT
ACACC+GGTGT
ACACG+CGTGT
ACACT+AGTGT
ACAGA+TCTGT

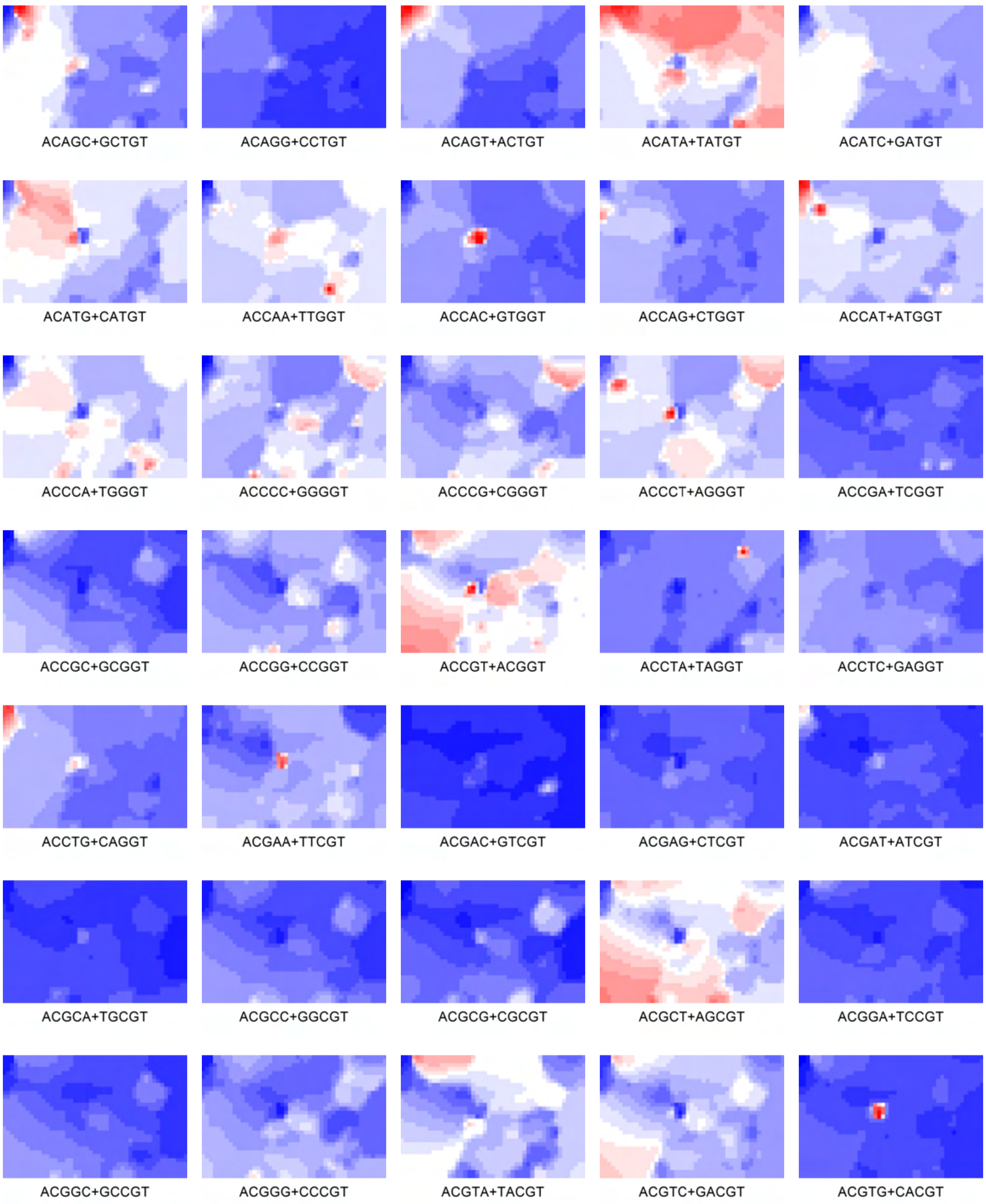

ACAGC+GCTGT
ACAGG+CCTGT
ACAGT+ACTGT
ACATA+TATGT
ACATC+GATGT
ACATG+CATGT
ACCAA+TTGGT
ACCAC+GTGGT
ACCAG+CTGGT
ACCAT+ATGGT
ACCCA+TGGGT
ACCCC+GGGGT
ACCCG+CGGGT
ACCCT+AGGGT
ACCGA+TCGGT
ACCGC+GCGGT
ACCGG+CCGGT
ACCGT+ACGGT
ACCTA+TAGGT
ACCTC+GAGGT
ACCTG+CAGGT
ACGAA+TTCGT
ACGAC+GTCGT
ACGAG+CTCGT
ACGAT+ATCGT
ACGCA+TGCGT
ACGCC+GGCGT
ACGCG+CGCGT
ACGCT+AGCGT
ACGGA+TCCGT
ACGGC+GCCGT
ACGGG+CCCGT
ACGTA+TACGT
ACGTC+GACGT
ACGTG+CACGT

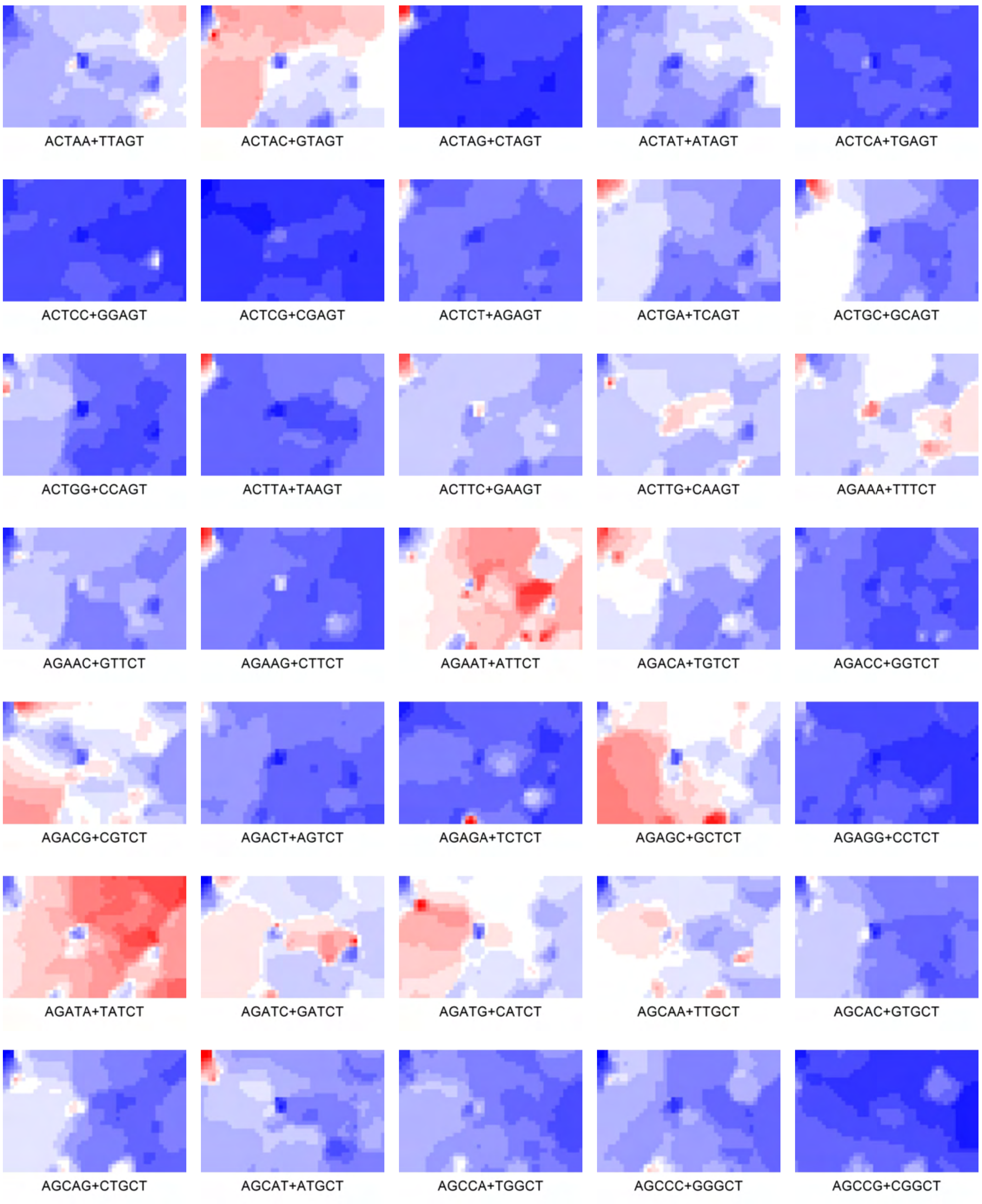
ACTAA+TTAGT
ACTAC+GTAGT
ACTAG+CTAGT
ACTAT+ATAGT
ACTCA+TGAGT
ACTCC+GGAGT
ACTCG+CGAGT
ACTCT+AGAGT
ACTGA+TCAGT
ACTGC+GCAGT
ACTGG+CCAGT
ACTTA+TAAGT
ACTTC+GAAGT
ACTTG+CAAGT
AGAAA+TTTCT
AGAAC+GTTCT
AGAAG+CTTCT
AGAAT+ATTCT
AGACA+TGTCT
AGACC+GGTCT
AGACG+CGTCT
AGACT+AGTCT
AGAGA+TCTCT
AGAGC+GCTCT
AGAGG+CCTCT
AGATA+TATCT
AGATC+GATCT
AGATG+CATCT
AGCAA+TTGCT
AGCAC+GTGCT
AGCAG+CTGCT
AGCAT+ATGCT
AGCCA+TGGCT
AGCCC+GGGCT
AGCCG+CGGCT

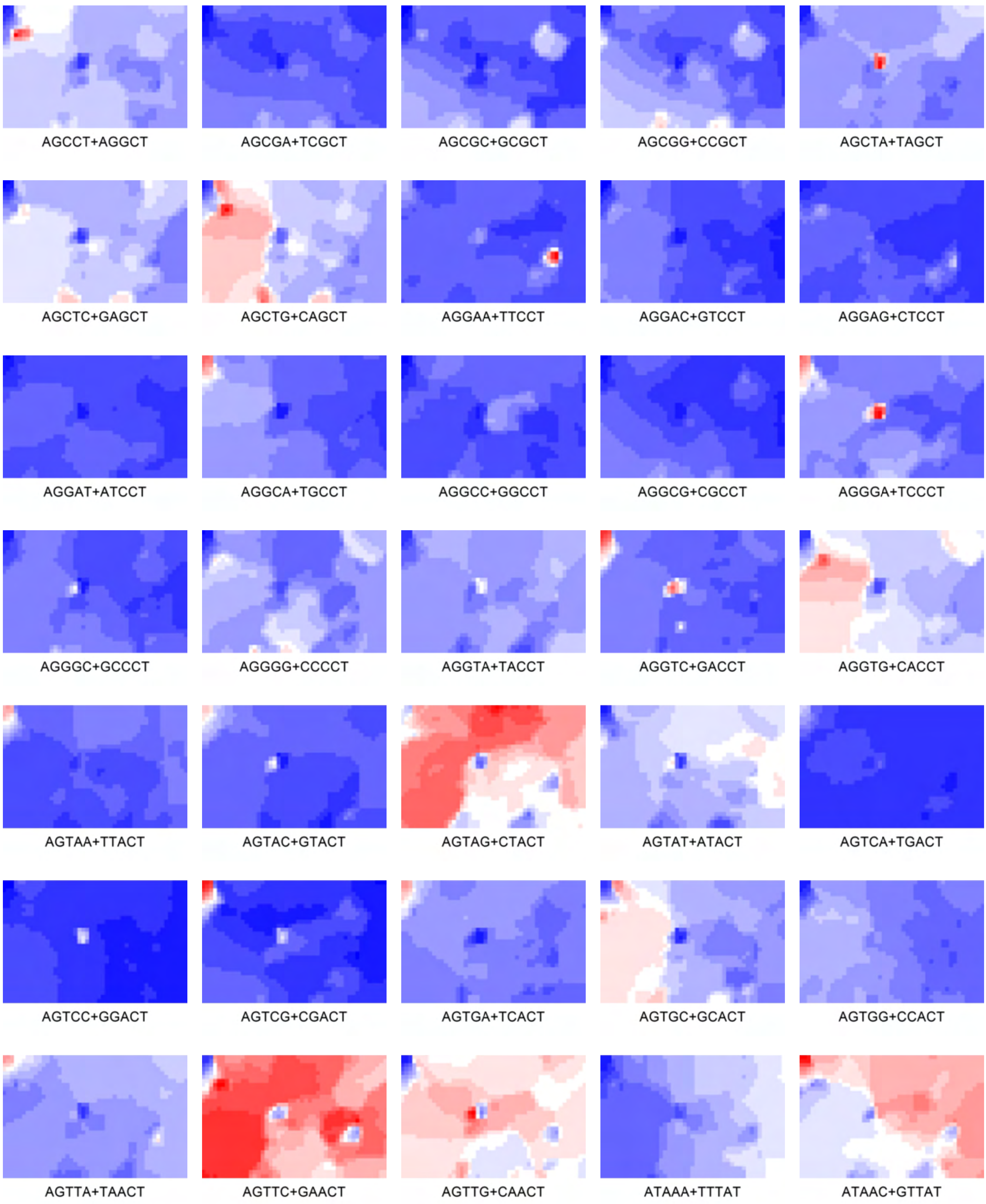
AGCCT+AGGCT
AGCGA+TCGCT
AGCGC+GCGCT
AGCGG+CCGCT
AGCTA+TAGCT
AGCTC+GAGCT
AGCTG+CAGCT
AGGAA+TTCCT
AGGAC+GTCCT
AGGAG+CTCCT
AGGAT+ATCCT
AGGCA+TGCCT
AGGCC+GGCCT
AGGCG+CGCCT
AGGGA+TCCCT
AGGGC+GCCCT
AGGGG+CCCCT
AGGTA+TACCT
AGGTC+GACCT
AGGTG+CACCT
AGTAA+TTACT
AGTAC+GTACT
AGTAG+CTACT
AGTAT+ATACT
AGTCA+TGACT
AGTCC+GGACT
AGTCG+CGACT
AGTGA+TCACT
AGTGC+GCACT
AGTGG+CCACT
AGTTA+TAACT
AGTTC+GAACT
AGTTG+CAACT
ATAAA+TTTAT
ATAAC+GTTAT

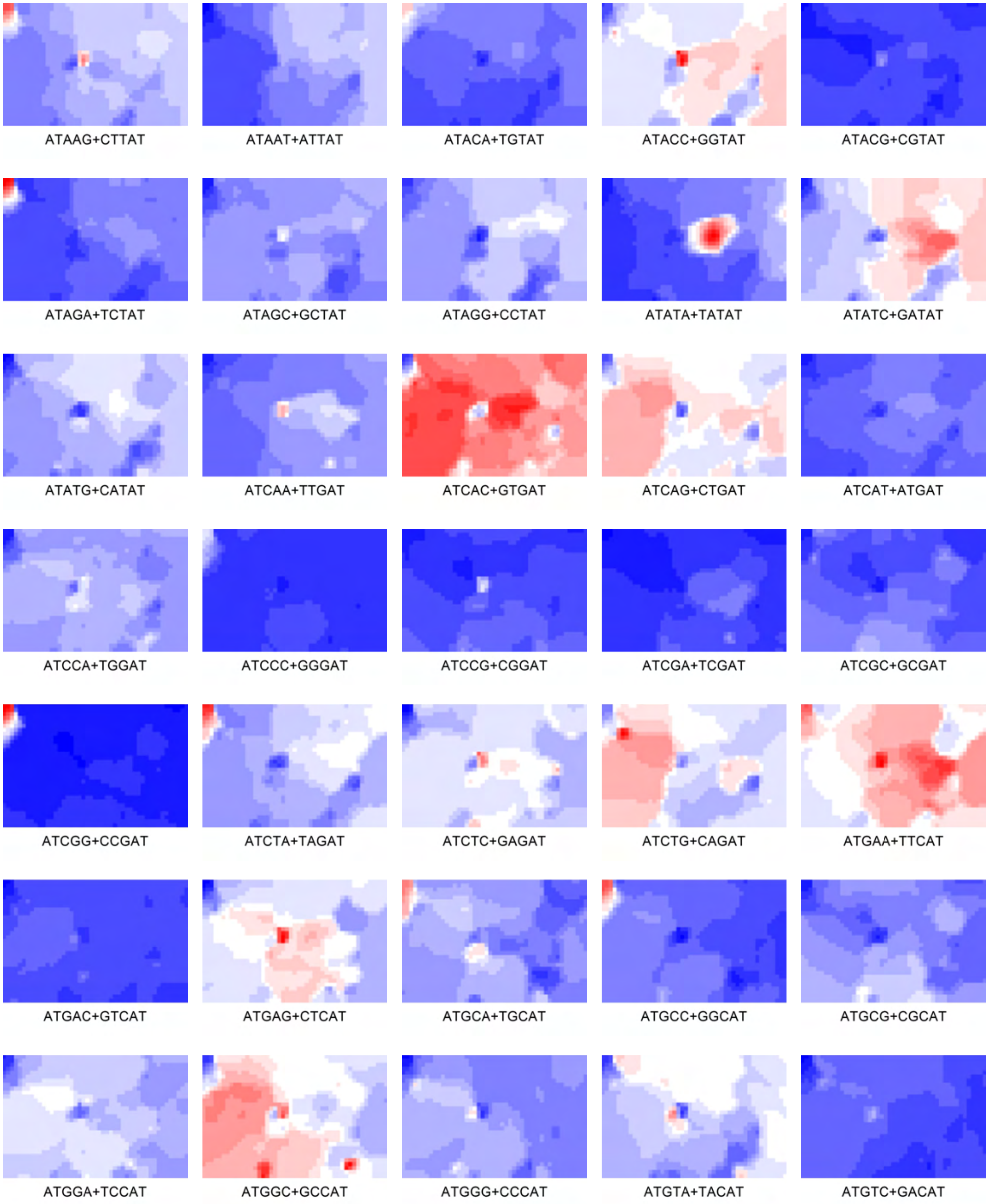
ATAAG+CTTAT
ATAAT+ATTAT
ATACA+TGTAT
ATACC+GGTAT
ATACG+CGTAT
ATAGA+TCTAT
ATAGC+GCTAT
ATAGG+CCTAT
ATATA+TATAT
ATATC+GATAT
ATATG+CATAT
ATCAA+TTGAT
ATCAC+GTGAT
ATCAG+CTGAT
ATCAT+ATGAT
ATCCA+TGGAT
ATCCC+GGGAT
ATCCG+CGGAT
ATCGA+TCGAT
ATCGC+GCGAT
ATCGG+CCGAT
ATCTA+TAGAT
ATCTC+GAGAT
ATCTG+CAGAT
ATGAA+TTCAT
ATGAC+GTCAT
ATGAG+CTCAT
ATGCA+TGCAT
ATGCC+GGCAT
ATGCG+CGCAT
ATGGA+TCCAT
ATGGC+GCCAT
ATGGG+CCCAT
ATGTA+TACAT
ATGTC+GACAT

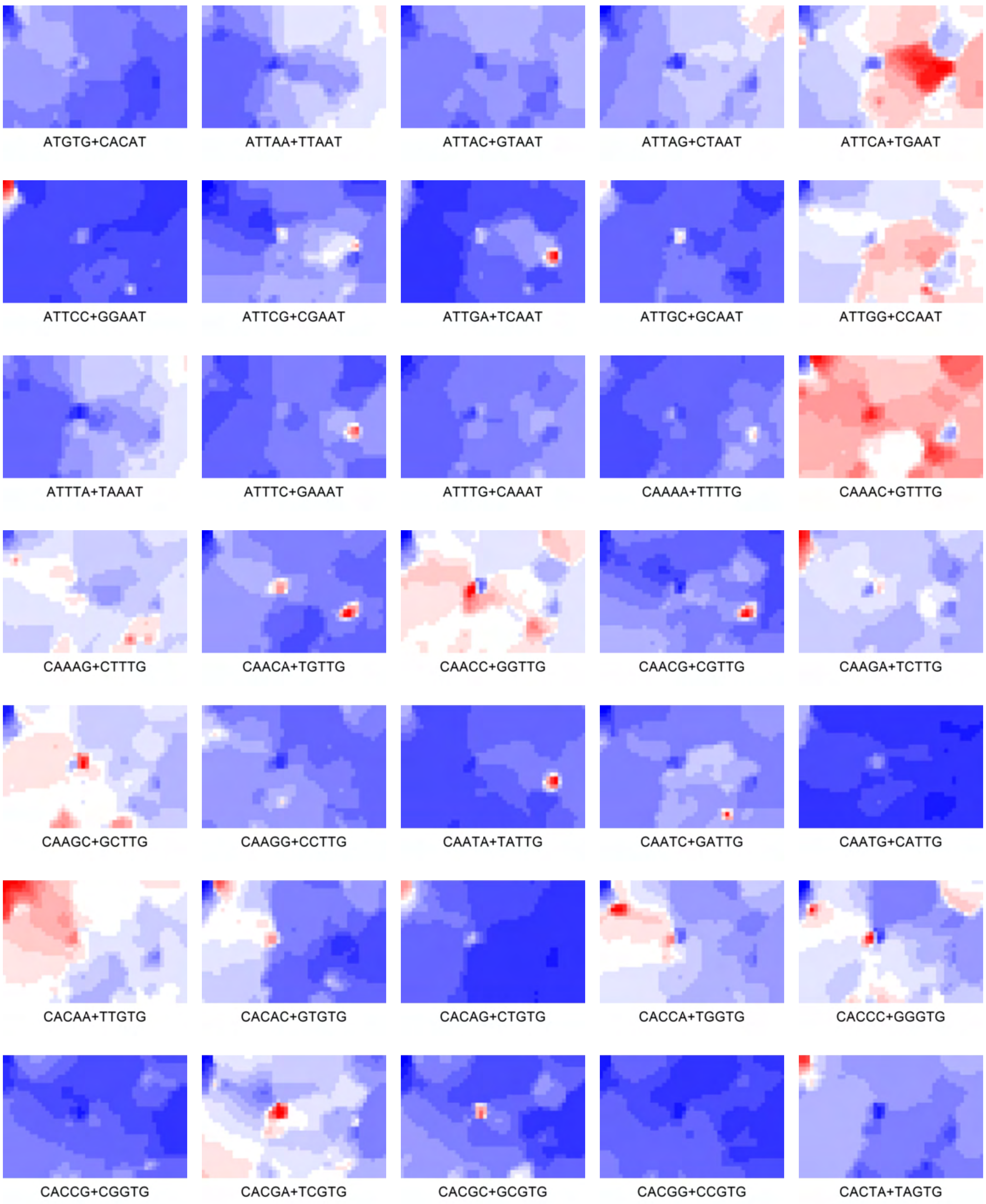
ATGTG+CACAT
ATTAA+TTAAT
ATTAC+GTAAT
ATTAG+CTAAT
ATTCA+TGAAT
ATTCC+GGAAT
ATTCG+CGAAT
ATTGA+TCAAT
ATTGC+GCAAT
ATTGG+CCAAT
ATTTA+TAAAT
ATTTC+GAAAT
ATTTG+CAAAT
CAAAA+TTTTG
CAAAC+GTTTG
CAAAG+CTTTG
CAACA+TGTTG
CAACC+GGTTG
CAACG+CGTTG
CAAGA+TCTTG
CAAGC+GCTTG
CAAGG+CCTTG
CAATA+TATTG
CAATC+GATTG
CAATG+CATTG
CACAA+TTGTG
CACAC+GTGTG
CACAG+CTGTG
CACCA+TGGTG
CACCC+GGGTG
CACCG+CGGTG
CACGA+TCGTG
CACGC+GCGTG
CACGG+CCGTG
CACTA+TAGTG

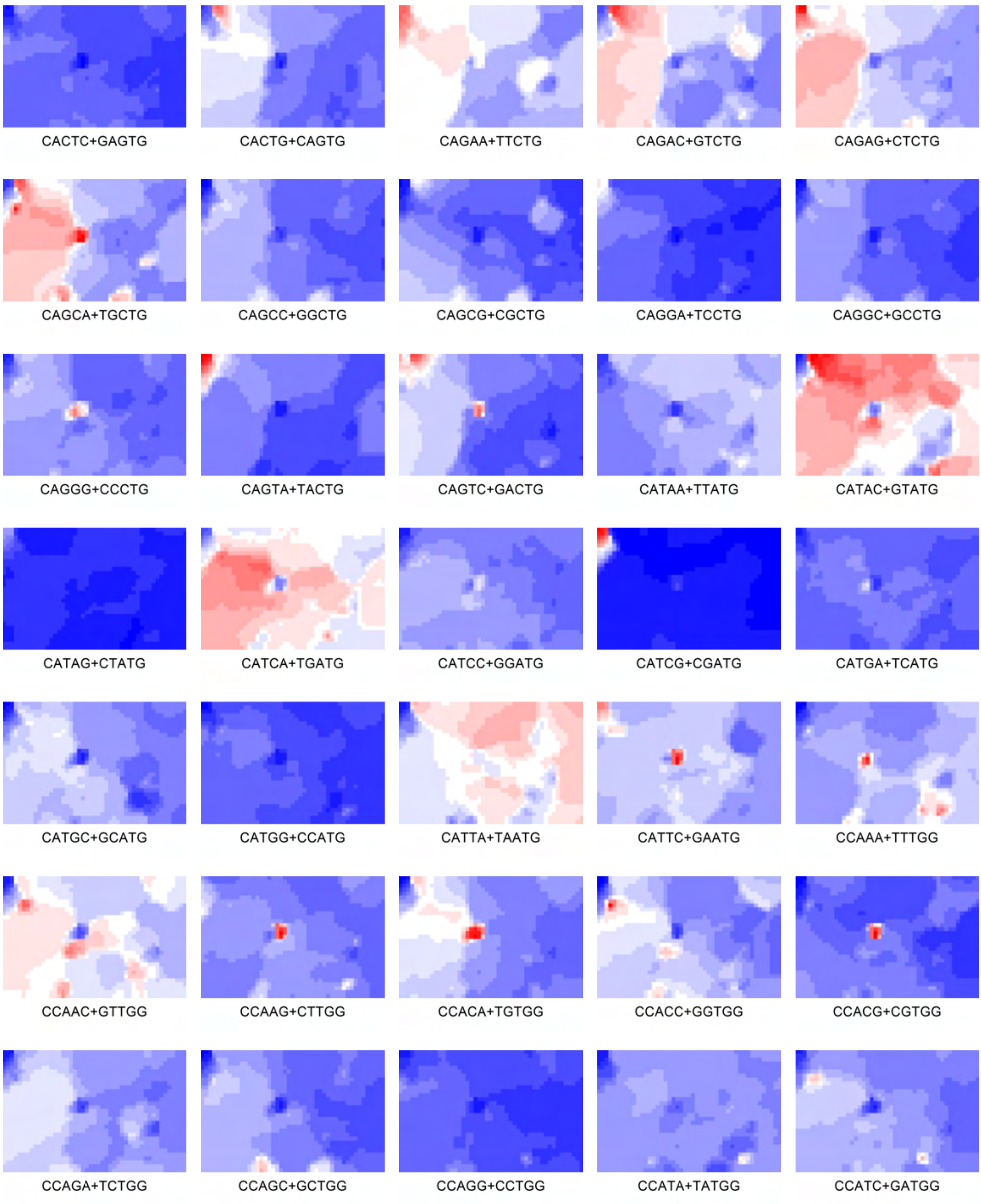

CACTC+GAGTG
CACTG+CAGTG
CAGAA+TTCTG
CAGAC+GTCTG
CAGAG+CTCTG
CAGCA+TGCTG
CAGCC+GGCTG
CAGCG+CGCTG
CAGGA+TCCTG
CAGGC+GCCTG
CAGGG+CCCTG
CAGTA+TACTG
CAGTC+GACTG
CATAA+TTATG
CATAC+GTATG
CATAG+CTATG
CATCA+TGATG
CATCC+GGATG
CATCG+CGATG
CATGA+TCATG
CATGC+GCATG
CATGG+CCATG
CATTA+TAATG
CATTC+GAATG
CCAAA+TTTGG
CCAAC+GTTGG
CCAAG+CTTGG
CCACA+TGTGG
CCACC+GGTGG
CCACG+CGTGG
CCAGA+TCTGG
CCAGC+GCTGG
CCAGG+CCTGG
CCATA+TATGG
CCATC+GATGG

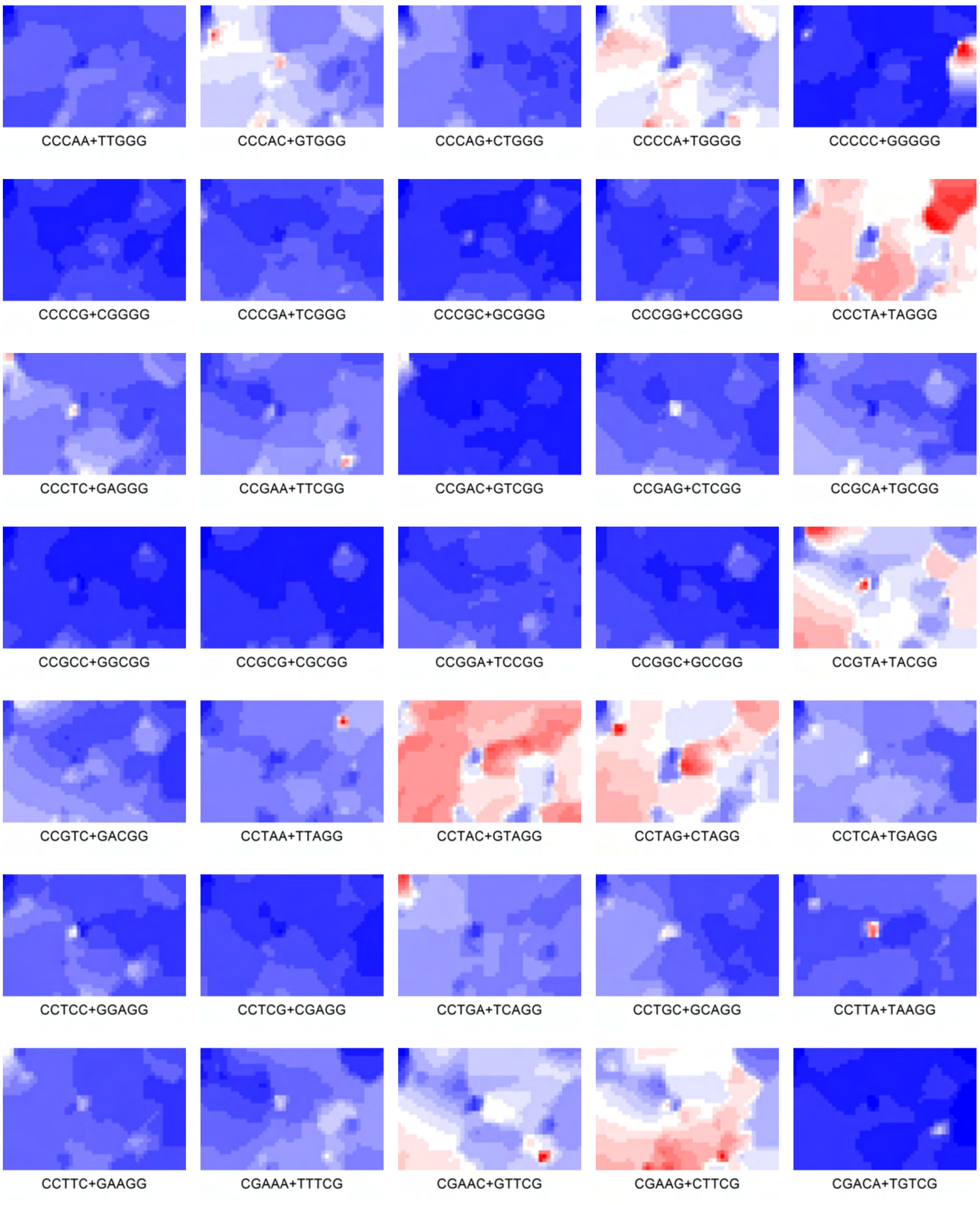
CCCAA+TTGGG
CCCAC+GTGGG
CCCAG+CTGGG
CCCCA+TGGGG
CCCCC+GGGGG
CCCCG+CGGGG
CCCGA+TCGGG
CCCGC+GCGGG
CCCGG+CCGGG
CCCTA+TAGGG
CCCTC+GAGGG
CCGAA+TTCGG
CCGAC+GTCGG
CCGAG+CTCGG
CCGCA+TGCGG
CCGCC+GGCGG
CCGCG+CGCGG
CCGGA+TCCGG
CCGGC+GCCGG
CCGTA+TACGG
CCGTC+GACGG
CCTAA+TTAGG
CCTAC+GTAGG
CCTAG+CTAGG
CCTCA+TGAGG
CCTCC+GGAGG
CCTCG+CGAGG
CCTGA+TCAGG
CCTGC+GCAGG
CCTTA+TAAGG
CCTTC+GAAGG
CGAAA+TTTCG
CGAAC+GTTCG
CGAAG+CTTCG
CGACA+TGTCG

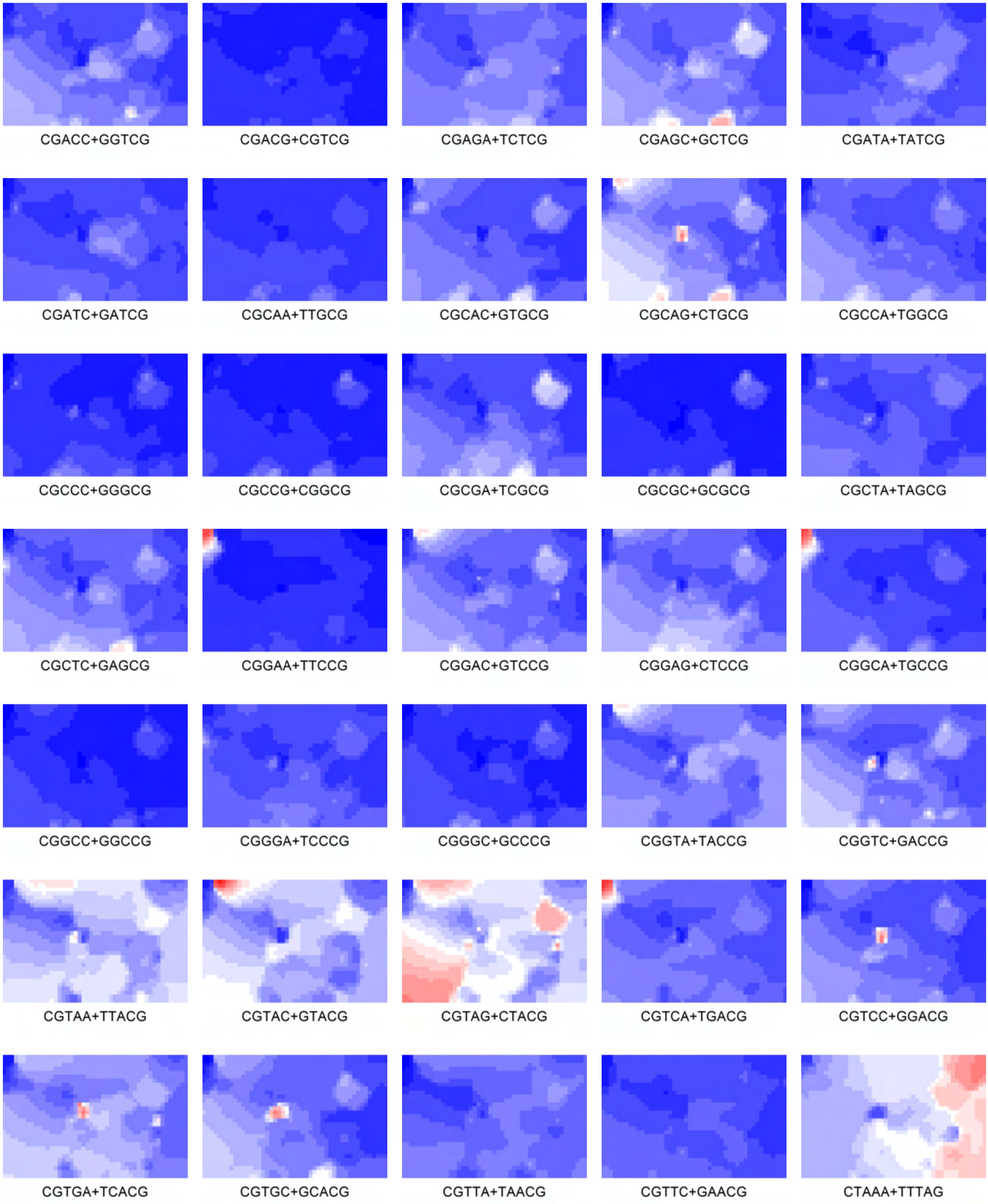
CGACC+GGTCG
CGACG+CGTCG
CGAGA+TCTCG
CGAGC+GCTCG
CGATA+TATCG
CGATC+GATCG
CGCAA+TTGCG
CGCAC+GTGCG
CGCAG+CTGCG
CGCCA+TGGCG
CGCCC+GGGCG
CGCCG+CGGCG
CGCGA+TCGCG
CGCGC+GCGCG
CGCTA+TAGCG
CGCTC+GAGCG
CGGAA+TTCCG
CGGAC+GTCCG
CGGAG+CTCCG
CGGCA+TGCCG
CGGCC+GGCCG
CGGGA+TCCCG
CGGGC+GCCCG
CGGTA+TACCG
CGGTC+GACCG
CGTAA+TTACG
CGTAC+GTACG
CGTAG+CTACG
CGTCA+TGACG
CGTCC+GGACG
CGTGA+TCACG
CGTGC+GCACG
CGTTA+TAACG
CGTTC+GAACG
CTAAA+TTTAG

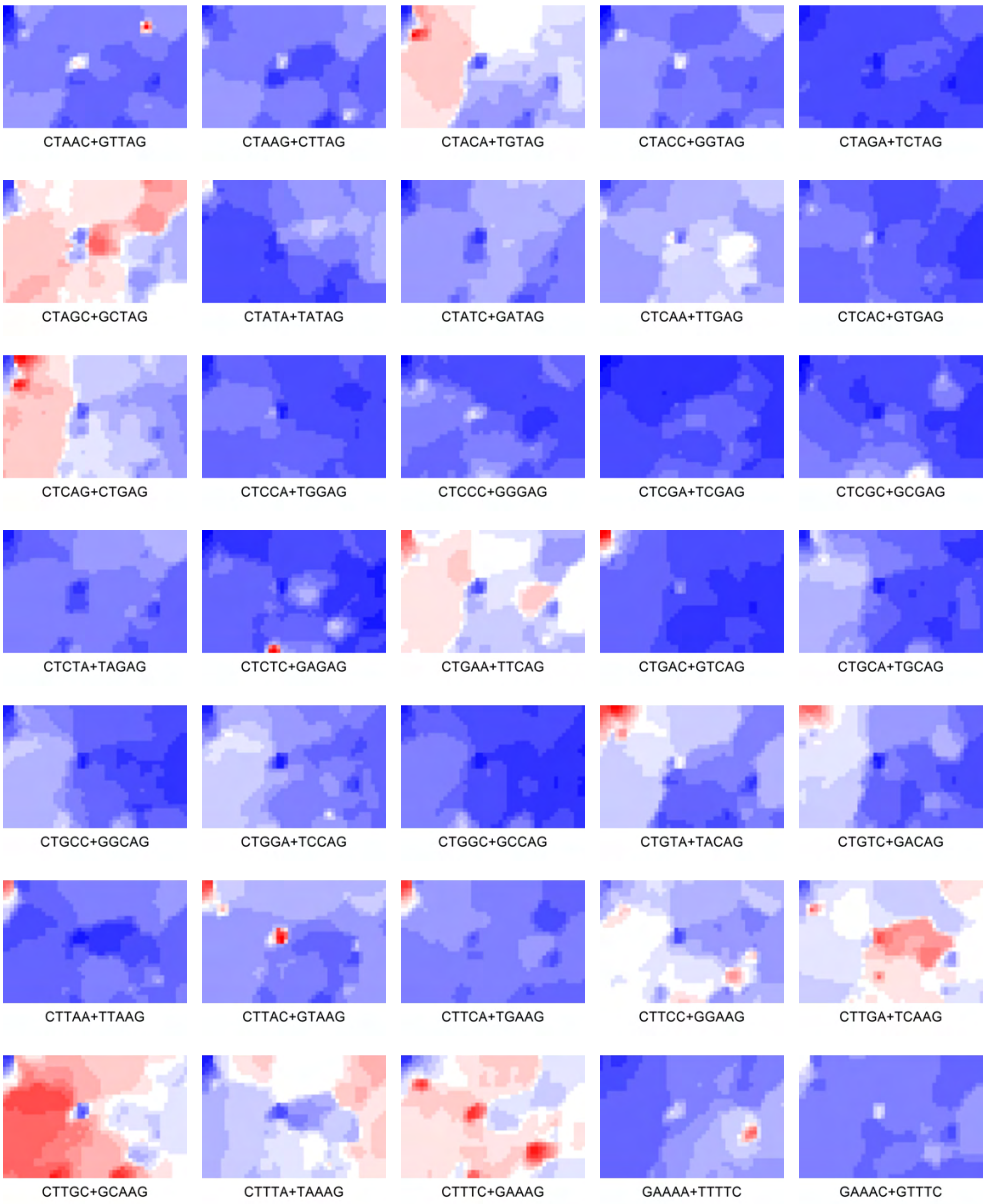
CTAAC+GTTAG
CTAAG+CTTAG
CTACA+TGTAG
CTACC+GGTAG
CTAGA+TCTAG
CTAGC+GCTAG
CTATA+TATAG
CTATC+GATAG
CTCAA+TTGAG
CTCAC+GTGAG
CTCAG+CTGAG
CTCCA+TGGAG
CTCCC+GGGAG
CTCGA+TCGAG
CTCGC+GCGAG
CTCTA+TAGAG
CTCTC+GAGAG
CTGAA+TTCAG
CTGAC+GTCAG
CTGCA+TGCAG
CTGCC+GGCAG
CTGGA+TCCAG
CTGGC+GCCAG
CTGTA+TACAG
CTGTC+GACAG
CTTAA+TTAAG
CTTAC+GTAAG
CTTCA+TGAAG
CTTCC+GGAAG
CTTGA+TCAAG
CTTGC+GCAAG
CTTTA+TAAAG
CTTTC+GAAAG
GAAAA+TTTTC
GAAAC+GTTTC

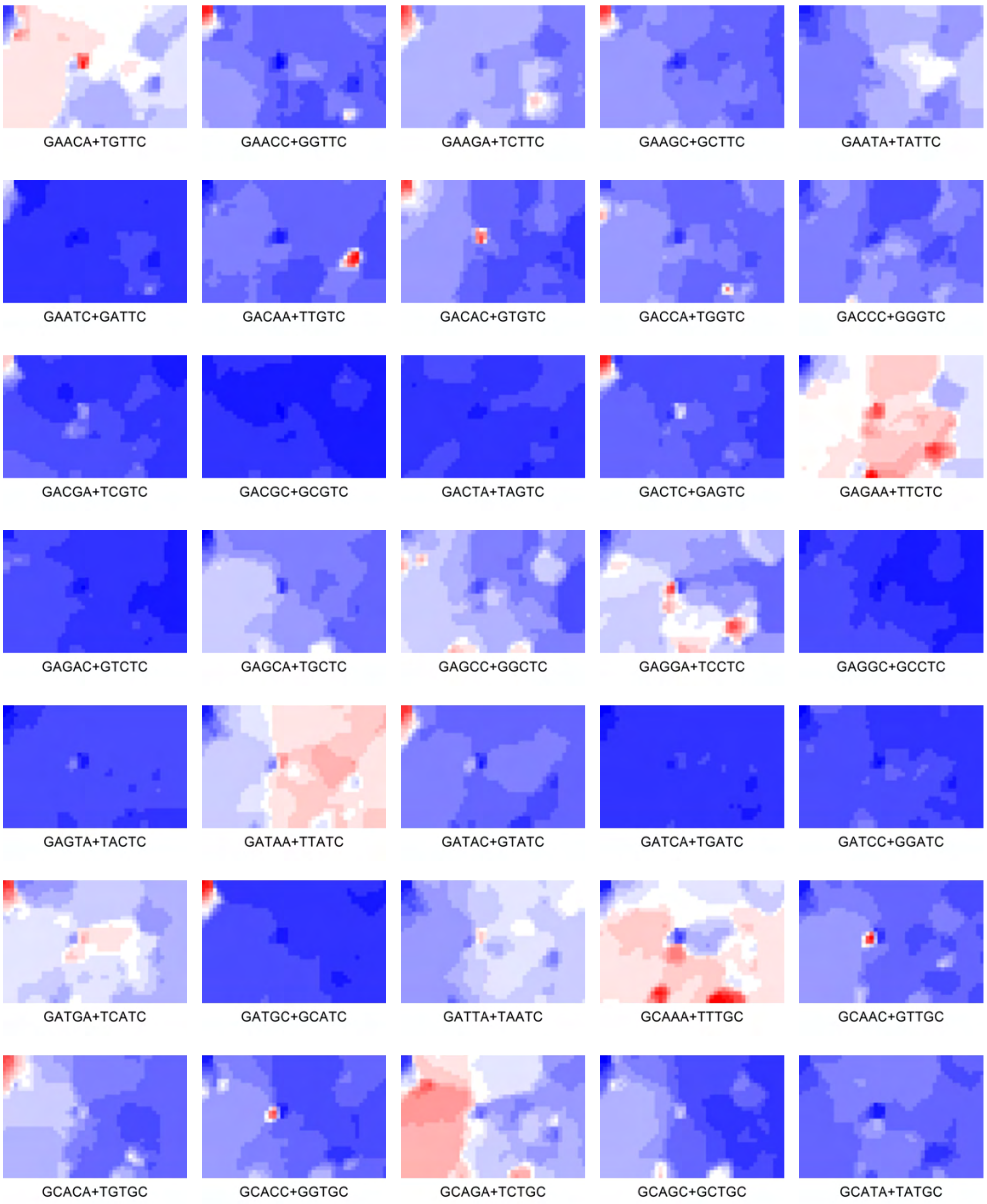
GAACA+TGTTC
GAACC+GGTTC
GAAGA+TCTTC
GAAGC+GCTTC
GAATA+TATTC
GAATC+GATTC
GACAA+TTGTC
GACAC+GTGTC
GACCA+TGGTC
GACCC+GGGTC
GACGA+TCGTC
GACGC+GCGTC
GACTA+TAGTC
GACTC+GAGTC
GAGAA+TTCTC
GAGAC+GTCTC
GAGCA+TGCTC
GAGCC+GGCTC
GAGGA+TCCTC
GAGGC+GCCTC
GAGTA+TACTC
GATAA+TTATC
GATAC+GTATC
GATCA+TGATC
GATCC+GGATC
GATGA+TCATC
GATGC+GCATC
GATTA+TAATC
GCAAA+TTTGC
GCAAC+GTTGC
GCACA+TGTGC
GCACC+GGTGC
GCAGA+TCTGC
GCAGC+GCTGC
GCATA+TATGC

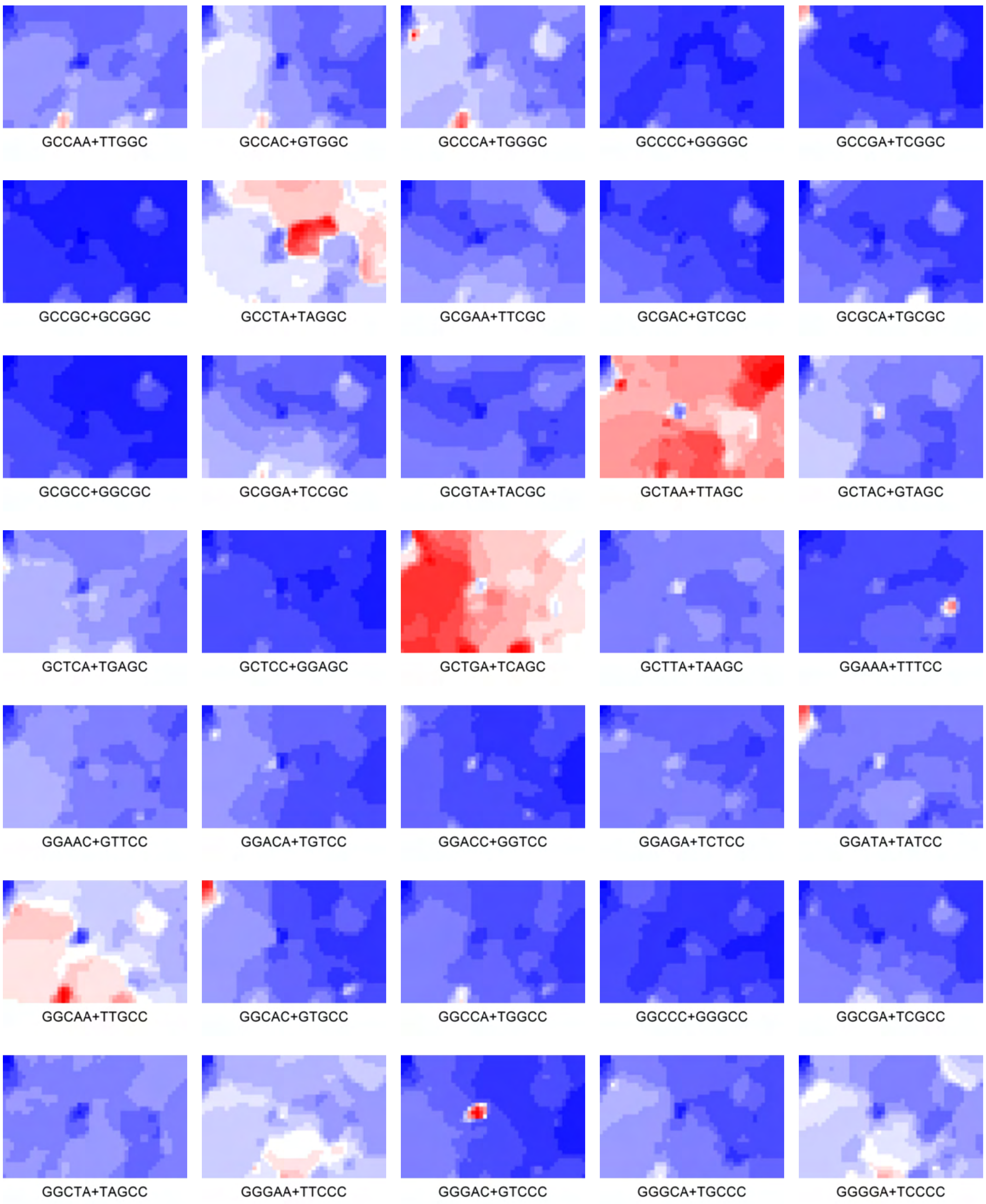
GCCAA+TTGGC
GCCAC+GTGGC
GCCCA+TGGGC
GCCCC+GGGGC
GCCGA+TCGGC
GCCGC+GCGGC
GCCTA+TAGGC
GCGAA+TTCGC
GCGAC+GTCGC
GCGCA+TGCGC
GCGCC+GGCGC
GCGGA+TCCGC
GCGTA+TACGC
GCTAA+TTAGC
GCTAC+GTAGC
GCTCA+TGAGC
GCTCC+GGAGC
GCTGA+TCAGC
GCTTA+TAAGC
GGAAA+TTTCC
GGAAC+GTTCC
GGACA+TGTCC
GGACC+GGTCC
GGAGA+TCTCC
GGATA+TATCC
GGCAA+TTGCC
GGCAC+GTGCC
GGCCA+TGGCC
GGCCC+GGGCC
GGCGA+TCGCC
GGCTA+TAGCC
GGGAA+TTCCC
GGGAC+GTCCC
GGGCA+TGCCC
GGGGA+TCCCC

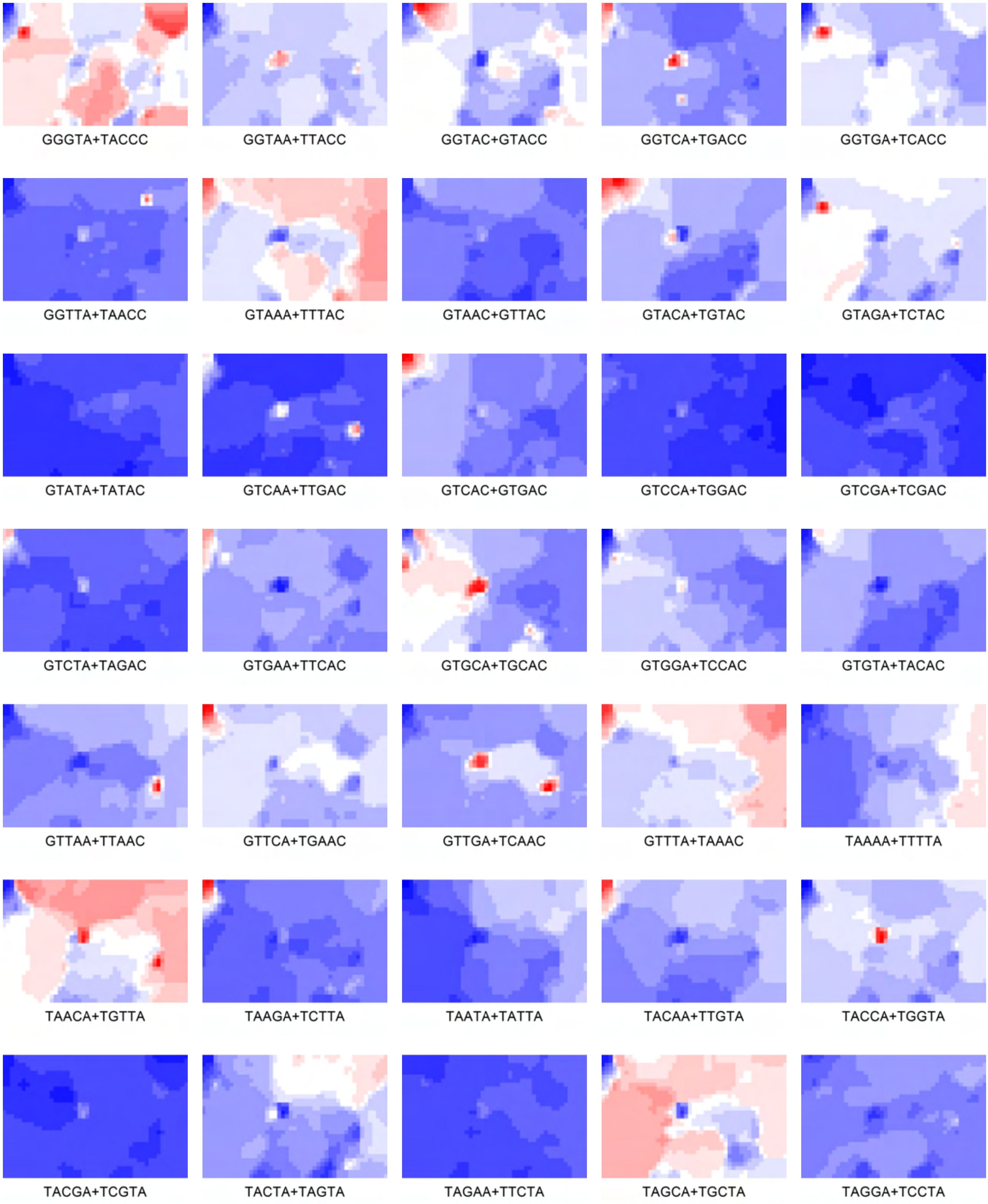
GGGTA+TACCC
GGTAA+TTACC
GGTAC+GTACC
GGTCA+TGACC
GGTGA+TCACC
GGTTA+TAACC
GTAAA+TTTAC
GTAAC+GTTAC
GTACA+TGTAC
GTAGA+TCTAC
GTATA+TATAC
GTCAA+TTGAC
GTCAC+GTGAC
GTCCA+TGGAC
GTCGA+TCGAC
GTCTA+TAGAC
GTGAA+TTCAC
GTGCA+TGCAC
GTGGA+TCCAC
GTGTA+TACAC
GTTAA+TTAAC
GTTCA+TGAAC
GTTGA+TCAAC
GTTTA+TAAAC
TAAAA+TTTTA
TAACA+TGTTA
TAAGA+TCTTA
TAATA+TATTA
TACAA+TTGTA
TACCA+TGGTA
TACGA+TCGTA
TACTA+TAGTA
TAGAA+TTCTA
TAGCA+TGCTA
TAGGA+TCCTA

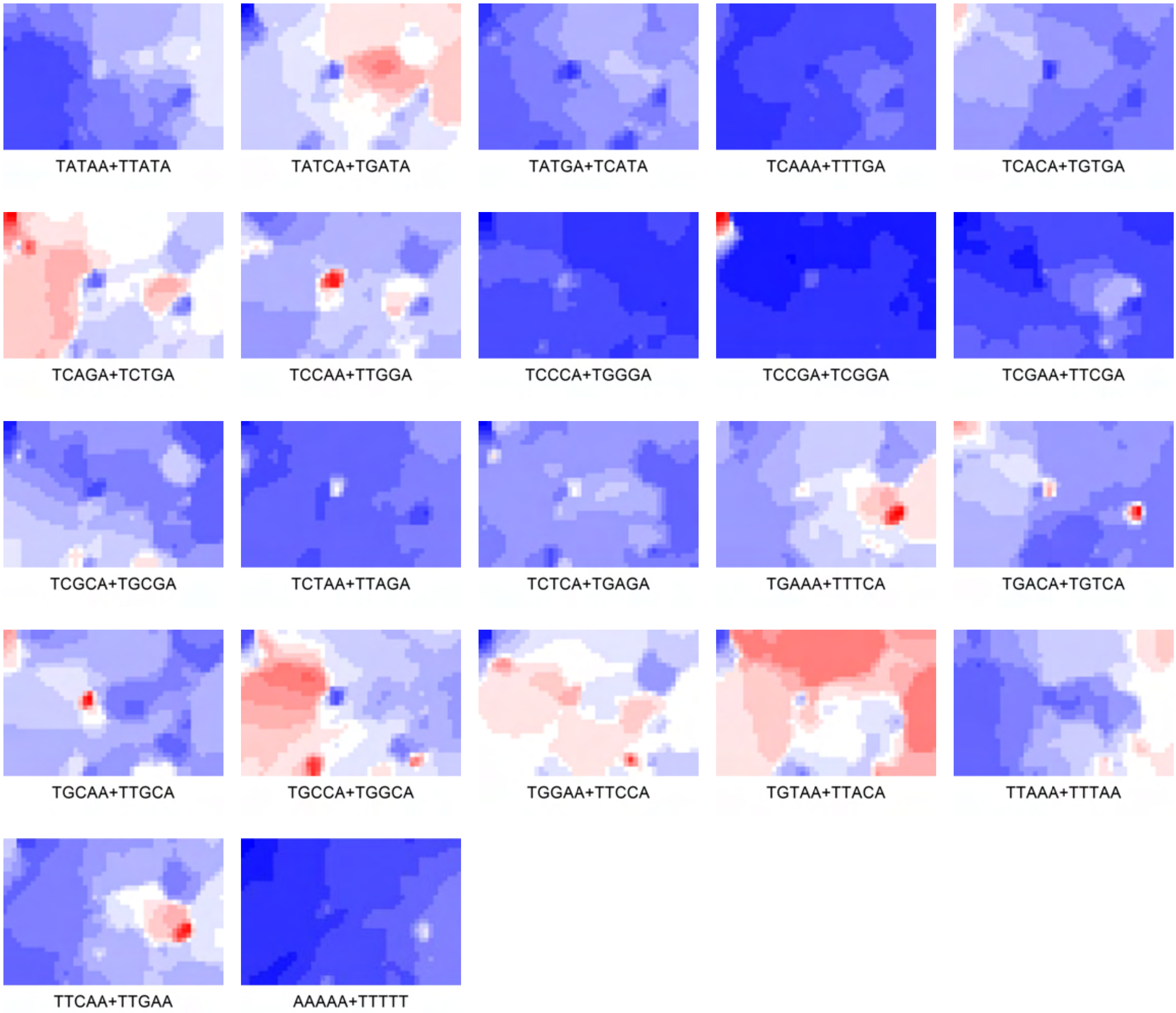
TATAA+TTATA
TATCA+TGATA
TATGA+TCATA
TCAAA+TTTGA
TCACA+TGTGA
TCAGA+TCTGA
TCCAA+TTGGA
TCCCA+TGGGA
TCCGA+TCGGA
TCGAA+TTCGA
TCGCA+TGCGA
TCTAA+TTAGA
TCTCA+TGAGA
TGAAA+TTTCA
TGACA+TGTCA
TGCAA+TTGCA
TGCCA+TGGCA
TGGAA+TTCCA
TGTAA+TTACA
TTAAA+TTTAA
TTCAA+TTGAA
AAAAA+TTTTT
